# Transparent neural interfaces: challenges and solutions of microengineered multimodal implants designed to measure intact neuronal populations using high-resolution electrophysiology and microscopy simultaneously

**DOI:** 10.1038/s41378-023-00519-x

**Published:** 2023-05-18

**Authors:** Z. Fekete, A. Zátonyi, A. Kaszás, M. Madarász, A. Slézia

**Affiliations:** 1 Research Group for Implantable Microsystems, Faculty of Information Technology & Bionics, Pázmány Péter Catholic University, Budapest, Hungary; 2 Institute of Cognitive Neuroscience & Psychology, Eotvos Lorand Research Network, Budapest, Hungary; 3 Mines Saint-Etienne, Centre CMP, Département BEL, F - 13541 Gardanne, France; 4 Institut de Neurosciences de la Timone, CNRS UMR 7289 & Aix-Marseille Université, 13005 Marseille, France; 5 János Szentágothai PhD Program of Semmelweis University, Budapest, Hungary; 6 BrainVision Center, Budapest, Hungary

**Keywords:** Optical materials and structures, Materials science

## Abstract

The aim of this review is to present a comprehensive overview of the feasibility of using transparent neural interfaces in multimodal in vivo experiments on the central nervous system. Multimodal electrophysiological and neuroimaging approaches hold great potential for revealing the anatomical and functional connectivity of neuronal ensembles in the intact brain. Multimodal approaches are less time-consuming and require fewer experimental animals as researchers obtain denser, complex data during the combined experiments. Creating devices that provide high-resolution, artifact-free neural recordings while facilitating the interrogation or stimulation of underlying anatomical features is currently one of the greatest challenges in the field of neuroengineering. There are numerous articles highlighting the trade-offs between the design and development of transparent neural interfaces; however, a comprehensive overview of the efforts in material science and technology has not been reported. Our present work fills this gap in knowledge by introducing the latest micro- and nanoengineered solutions for fabricating substrate and conductive components. Here, the limitations and improvements in electrical, optical, and mechanical properties, the stability and longevity of the integrated features, and biocompatibility during in vivo use are discussed.

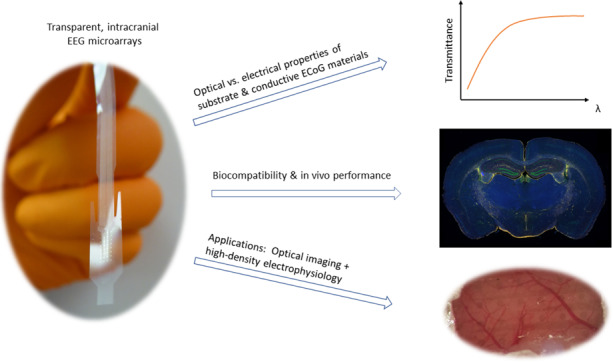

## Introduction

Neuroimaging methods are extensively combined with other experimental techniques in the field of neuroscience to study neural activity and map brain connectivity^1^. This combined approach allows brain mechanisms and pathological states to be understood and contributes to the identification of biomarkers for neurological diseases. Among the techniques used for monitoring brain activity, electrophysiology is still the gold standard. Recording electrodes can be placed noninvasively on the scalp (electroencephalography, EEG) or invasively inside the skull (electrocorticography, ECoG). Invasive recording methods have two fundamentally different approaches: penetrating probes that record from a single neuron or a small population of neurons^2^ or flexible and soft electrode arrays on the surface (epidural) or under (subdural) the dura mater that record network activity^3^. Although ECoG devices are invasive probes that require microsurgical implantation, they offer better signal quality, longevity, reliability, and spatial and temporal resolution and cover a wider frequency band than EEG devices^4^. ECoG devices have a strong track record in clinical applications, especially in epilepsy surgery^5,6^, and have also been demonstrated to control brain–computer interfaces^7^. The spatial information related to brain signals provided by ECoG devices is affected by the size of the recording sites. Although current photolithography techniques may provide sufficient spatial resolution for defining micro- and nanoscale conductive features, the deterioration of the signal-to-noise ratio upon miniaturization is inevitable.

**Fig. 1:**
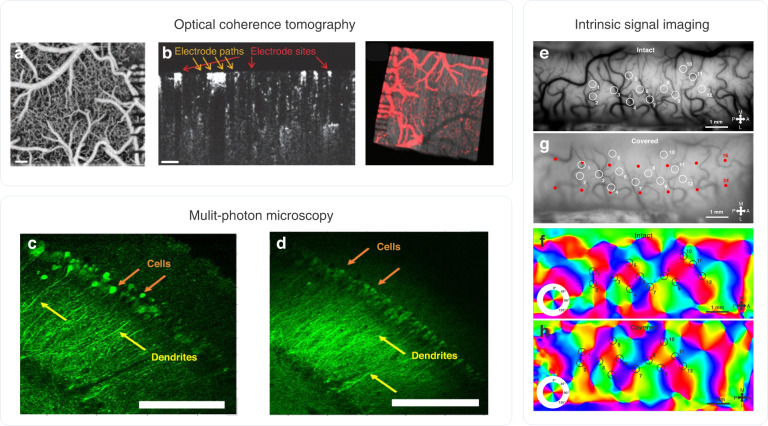
Optical coherence tomography. **a** Maximum intensity projection of an OCT angiogram showing cortical vasculature visible through a Parlyene C/graphene-based ECoG device. **b**, left Cross-sectional angiogram. **f**, right 3D visualization of the vasculature (red color) overlaid on the structural data (gray). Scale bars, 200 microns (**a**), 100 microns (**b**). Two-photon microscopy. Fluorescent images of CA1 neurons resolved in hippocampal slices not covered (**c**) and covered (**d**) with the transparent, Parylene HT/ITO-based ECoG using 72 and 22.3 mW power of two-photon excitation, respectively. The red and yellow arrows indicate cell soma and dendrites, respectively. Intrinsic signal imaging. **e** Vascular images of the exposed region of cat visual cortex A18 uncovered (**e**) and covered with a transparent polyimide/ITO-based ECoG electrode array (**g**). Orientation maps recorded using optical intrinsic signal imaging in each case are represented by (**f**) and (**h**). White and black circles with numbers and red dots show pinwheels and recording site positions of the electrode, respectively. Each numbered circle refers to the position of the same pinwheel on each picture. All images are reproduced with permission from the work of refs. ^8–10^

**Fig. 2:**
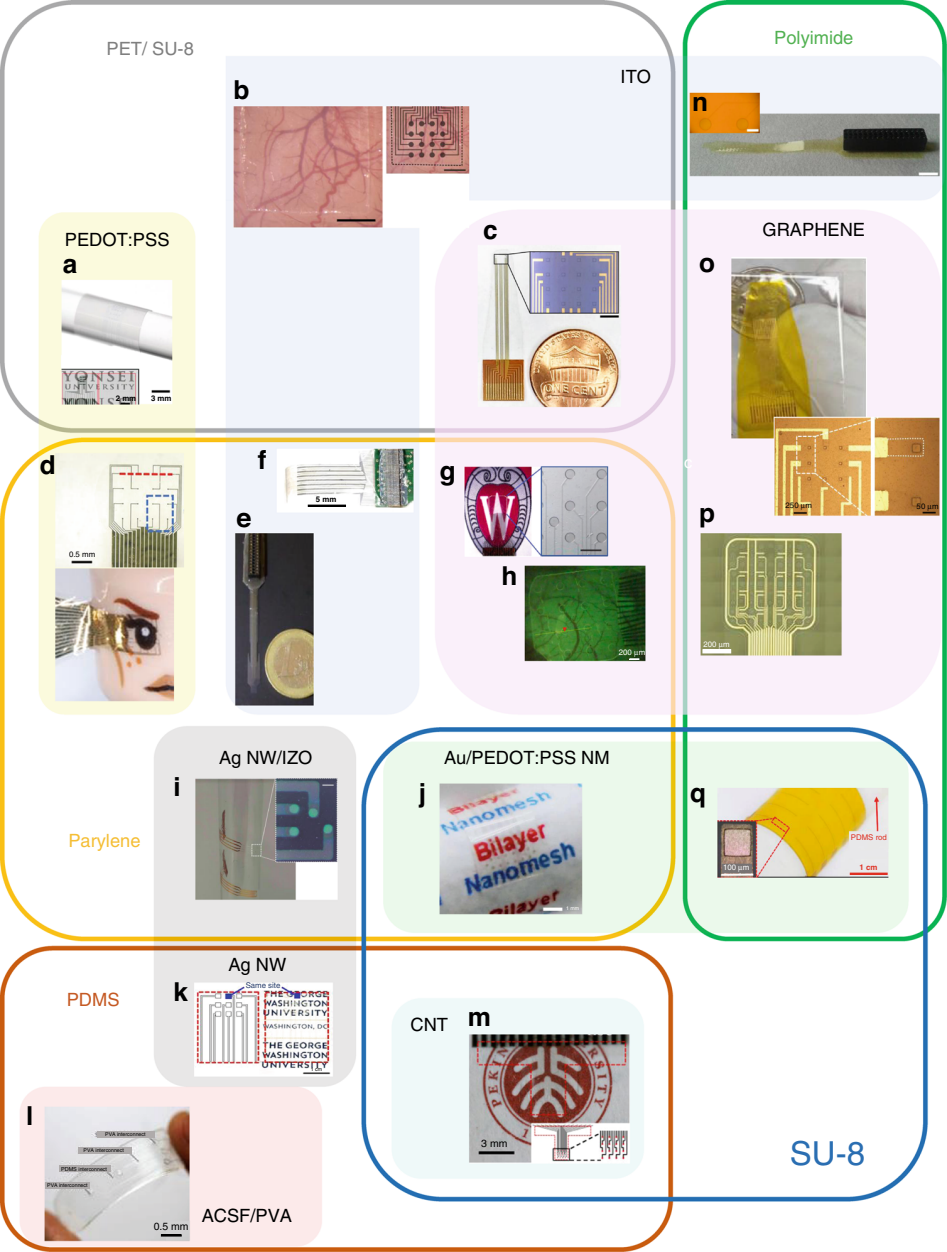
Examples of transparent neurointerface devices grouped by layer structures. **a**) PET/PEDOT:PSS/SU-8: ref. ^34^, **b**) PET/ITO/SU-8: ref. ^25^, **c**) PET/graphene/SU-8: ref. ^29^, **d**) PaC/PEDOT:PSS: ref. ^31^, **e**) PaHT/ITO ref. ^9^, **f**) PaC/ITO ref. ^21^, **g**) PaC/graphene ref. ^8^, **h**) PaC/graphene ref. ^28^, **i**) PaC/Ag NW/IZO ref. ^36^, **j**) PaC/Au/PEDOT:PSS/SU-8: ref. ^85^, **k**) PDMS/AG NW: ref. ^57^, **l**) PDMS/ACSF/PVA: ref. ^37^, **m**) PDMS/CNT/SU-8: ref. ^30^, **n**) PI/ITO ref. ^10^, **o**) Polyimide/graphene/polyimide: ref. ^24^, **p**) PI/graphene: ref. ^187^, **q**) PI/Au/PEDOT:PSS/SU-8: ref. ^51^. All figures are reproduced with permission

Despite advances in photonics, biochemistry, and molecular genetics, the limitations of individual neuroimaging techniques have compelled neuroscientists to combine multiple approaches, such as electrophysiology with functional magnetic resonance imaging, multiphoton imaging, or optogenetics. Neuroimaging techniques can provide desired spatial information that complements the information obtained from ECoG. Intrinsic optical signal imaging (IOSI), optical coherence tomography (OCT), multiphoton calcium imaging, and voltage-sensitive dye imaging (VSDI) are the most popular means of optically studying cortical activity with outstanding spatial resolution^1^. Figure 1 shows practical examples of utilizing transparent microdevices to carry out in vivo electrophysiology in combination with OCT^8^, two-photon^9^, and IOSI recordings^10^. These experiments clearly show that different types of illumination may facilitate the use of various ECoG materials. For instance, the effect of popular transparent conductive layers (e.g., graphene and indium-tin-oxide) on image quality during intracortical scanning with a coherent laser source was thoroughly characterized and showed the limitations of material science approaches when collecting light reflected from blood cells (Fig. 1a) or detecting the fluorescent emission of labeled cells (Fig. 2b). IOSI recording with a polyimide-based device (Fig. 1c) also highlighted that even substrate materials with limited transparency can still be efficiently used in the beam path if incoherent light sources are applied. Even though the optical recordings are slightly compromised due to the presence of the transparent ECoG devices, the images are suitable for the evaluation of the vasculature (Fig. 1a), fluorescent calcium signals (Fig. 1b), and mapping of the functional domains in the cortex (Fig. 1c) and can provide further information on electrophysiological signals at the network level.

IOSI is an imaging technique that relies on reflectance changes in the brain in response to neuronal activation (Fig. 1c). The exposed surface of the cortex is illuminated with incoherent red light through a cranial chamber, and images derived from the reflection of light are recorded by a charged coupled device (CCD) camera. Hemodynamic responses of cortical regions to sensory stimulation outline active and inactive regions through changes in the degree of reflection, so the amount of light reflected is indirectly correlated with neuronal firing. IOSI has been used to map the organization and functional architecture of cortical regions in several species. It is often applied as a primary method for targeted electrophysiological or pharmacological intervention. It has a relatively high spatial resolution (~100 µm), and events as close as 100 ms can be resolved^11^. The advantage of IOSI is that vast cortical regions can be imaged label-free and repeatedly over a long period of time, with the downside being the indirectness of the signal and its long temporal delay^12,13^.

OCT imaging, such as ultrasound B-mode imaging, is a technique involving the detection of the echo time delay of backscattered light. Contrary to IOSI, where photons are directly measured, OCT relies on low-coherence interferometry. In this way, the back-scattering properties of different cortical layers can be measured. As a result, tissue morphology is exposed with a spatial resolution similar to conventional histology, even in vivo, without any excision or processing. OCT has a high lateral and axial resolution (1–10 µm), megahertz-rate temporal resolution (A-scan rate), and 1–4 mm penetration depth^14^. Functional OCT also enables label-free detection of hemodynamic and metabolic changes in the brain, including experiments that reveal developmental changes in various animal models.

Fluorescence microscopy, most notably two-photon laser scanning microscopy, is a widely used technique for examining intact brain tissue^15^ (Fig. 1a, b). The main difference between one-photon and two-photon microscopy is the physical process of light absorption, with the latter method’s process exciting much smaller tissue volumes using much lower energy at near-infrared wavelengths. With the use of near-infrared excitation with multiple photons, lower scattering, and higher penetration depths can be achieved, in addition to reduced out-of-focus excitation. However, excitation depends on the structure of the molecule excited, and many dyes are incompatible with multiphoton imaging due to their insufficient absorption cross-sections^16^.

The activity of cells can be visualized with fluorescent dyes (fluorophores). These molecules absorb a particular wavelength band of light (excitation wavelength) and then emit light at another, longer wavelength (emission wavelength). Live cells can be labeled by local dye injection into a tissue or by the expression of genetically encoded fluorescent proteins. Calcium indicators are the most common, which allow the determination of neuronal activity indirectly, increasing fluorescence with the increase in intracellular calcium concentration. While calcium imaging is the method of choice for measuring the activity of single neurons and populations in vivo, it is afflicted by the slow kinetics of the dyes and the influence of reporters on signal integration due to the binding of calcium ions.

VSDI is an invasive method that uses voltage-sensitive dyes to study the dynamics of cortical processing at high spatial (<50 microns) and temporal (1–10 ms) resolution^6^ over a large field of view (between 1–2 cm^2^). Dye molecules can be applied to the exposed cortex (without dura mater) to bind directly to the surface of the cell membrane or, alternatively, genetically encoded voltage indicators with viral vector injections. VSDs act as transducers that transform membrane potential changes into optical signals without altering physiological functionality. Upon excitation, emitted fluorescence is detected with a CCD camera. This fluorescent signal is proportional to the membrane area of labeled structures. Compared to calcium indicators, VSDs have kinetics that are significantly faster (ms range), but they also have a lower signal-to-noise ratio and are more sensitive to photobleaching^17^.

Neural interfaces combining ECoG and neuroimaging need to satisfy several requirements^18^. As neuroscience shifts increasingly toward chronic experiments on awake and behaving subjects, an ideal transparent neural interface needs to satisfy the demands of those experiments in terms of appropriate structures and materials. An ideal ECoG system that enables concurrent multimodal neuroimaging should be fully transparent both in its substrate and conductive layers so that it in no way optically hinders the chosen method of neuroimaging. It should be flexible and biocompatible, elicit no immune response and cause no physical damage due to movement after implantation. It should also be durable and highly tensile and tear-resistant, easing the process of implantation and preventing the loss of recording channels due to damage or over time. The manufacturing technology and process should be inexpensive and easy to access, adapt and produce, thereby supporting widespread use, large sample sizes, and the reproducibility of results. The recording sites should have low impedance, and the conductive material should be insensitive to artifact generation. It should accommodate the desired number, size, and pattern of recording sites with the smallest possible overall size. Finally, it should require little to no space on an animal’s head for signal transmission after implantation, allowing it to be placed anywhere without being an obstacle. Arguably, the ideal transparent neural interface should be tailored to the neuroimaging method of choice, as fundamental differences necessitate specific design choices for optimal performance, as well as to the experiment itself, whether awake or chronic functionality is needed. While many of these properties are still being researched, existing solutions have already proven successful in terms of achieving some of these ideal characteristics.

The validation of ECoG devices as tools compatible with optical methods emerged in the early 2010s. As a secondary factor in addition to flexibility, transparency has become a characteristic to strive for as well when developing novel devices. Optogenetic modulation (refs. ^19–21^), two-photon imaging of vascular growth^22,23^ and calcium^24^, VSD imaging^25^ and OCT^8^ were all used in conjunction with electrocorticography to prove the usability of the respective devices as promising tools for further research. Parylene C was favored as the substrate material, along with polyimide and polyethylene terephthalate (PET), while the electrode materials were more diverse: gold, indium-tin-oxide (ITO), and graphene were used in the process of optimizing transparency and electric properties. The second half of the decade saw the introduction of poly(3,4-ethylenedioxythiophene):poly(styrenesulfonate) (PEDOT:PSS), gold nanowires, and carbon nanotubes in transparent ECoG devices and the development of better graphene devices^26–29^. The methods and experiments used to emphasize the capabilities of these devices have also undergone development. Transparent multimodal devices were used to observe evoked seizure events^30,31^ and visually evoked potentials^32^, to functionally map the cat cortex with IOSI^10^ and to correlate ECoG signals with calcium and single-unit activity^33^. In the last several years, these transparent, multimodal devices have attracted considerably increasing attention. On the one hand, this attention was focused on novel material combinations, such as Parylene HT^9^, treated PEDOT:PSS-EG^34^, silver nanowires (refs. ^35,36^), polyvinyl-alcohol based hydrogel^37^ and thiol-ene/acrylate–iridium oxide^38^, and on deeper integration with current trends in neuroscience, such as gradient-index optics (GRIN) lenses, experiments with freely moving animals,^39^, mesoscale imaging^40^ and brain organoids^41^. On the other hand, established materials and fabrication techniques now enable the shift from development to experiment. Recently, ECoG devices have been used to deepen the understanding of the microscale spatiotemporal dynamics of seizures^42^, the patterns of corticohippocampal interactions^43^, and the correlation of sharp-wave ripples and hippocampal cell assemblies^44^, which now underlines, rather than validates, the place of these multimodal devices among current neuroscientific methods.

The aim of this review is to give an overview of transparent substrates and conductive materials that may provide a solution for fully exploiting both optical imaging and electrophysiology, focusing on transparent devices that enable chronic measurements of awake animals. Transparent conductive and substrate materials, their relevant properties and challenges in microfabrication, together with their effect on in vivo performance, are discussed. Sources of artifact generation and strategies for alleviating them are detailed, as well as available data on the biocompatibility of materials. Finally, we consider the application perspectives of multimodal, transparent neurointerfaces.

## Relevant properties of transparent neurointerface materials

The material composition of transparent neurointerfaces has a dominant influence on the quality of bioelectric and optical signals in a multimodal neuroimaging experiment. Conductive and substrate materials, preferably both, should be optically clean and transparent, while the resistivity of the conductive layer and sensitivity to exposed light should be as low as possible. In addition to a high signal-to-noise ratio (low site impedance) and completely artifact-free recordings, mechanical stability during implantation and in long-term use is also essential. Packaging is also a crucial factor, which may limit applicability in chronic (freely moving) conditions. The extent of distortion or attenuation of the optical signal due to the presence of the layer-stack is also important but rarely characterized. There are several conductive and substrate materials detailed in this review that have been proposed to meet at least some of these demands; however, a perfect solution has yet to be attained due to challenges in material synthesis and technology. This section provides a brief guide for readers to understand the most important properties and limitations of materials currently used in transparent microimplants specifically designed for intracranial EEG recording that provide a clean window to image or stimulate tissue with optical methods.

### Conductivity

In principle, an ideal, transparent intracranial interface should affix invisible and highly conductive traces and recording sites on the substrate, providing the maximum field of view for imaging applications while sampling field potentials at a high signal-to-noise ratio. Unfortunately, none of the currently used conductive implant materials can fulfill all of these requirements. Material engineers are developing new approaches in synthesis and technology to overcome this limitation. To characterize the electrical property of potential conductive layers, resistivity, defined in Eq. 1, can be used, which is the reciprocal of conductivity.

1 $$\rho = \frac{{RA}}{l} = \frac{1}{\sigma }$$

where R is the electrical resistance of the specimen made of the conductive material, A and l are its cross-sectional area and length, respectively, and σ is the conductivity.

In some of the literature, sheet resistance characterized by four-point probe measurements is also presented (Eq. 2.):

2 $$R_S = \frac{\rho }{t}$$

where R_S_ is the sheet resistance and t is the thickness of the characterized material.

Recently, impedance or normalized impedance has been used more frequently than resistivity and sheet resistance. This value is derived by measuring the complex impedance of the individual recording sites of the final device either in saline or in vivo using electrochemical impedance spectroscopy. This measurement takes all components of the equivalent circuit models of electrodes; therefore, the average of the absolute value of each site impedance measured at 1 kHz yields more informative data on the performance of the electrodes. Normalizing the impedance to the recording site area is also common.

An overview of the electrical and optical properties of conductive materials typically used in neuroengineering applications is given in Table 1. Apparently, the desired values of resistivity are accompanied by lower transparency, which leads to a necessary trade-off in material selection. In particular, the conventionally applied thin film metal layers with excellent conductive properties and mature technology, such as gold, platinum, iridium, and stainless steel, cannot be considered for use at all. Instead, inorganic (metal oxides and metal nanowires) and organic (carbon nanotubes, graphene, transparent conductive polymers) materials can be utilized. In general, high charge carrier densities and mobilities are desired to form a highly conductive layer. Charge carrier densities can be tailored by doping, which is a common strategy for facilitating the use of nonmetallic films in transparent electrodes. It should be noted that doping also has an impact on the sensitivity of the electrode to the photoelectric effect by reducing the photoelectric work function at higher doping concentrations.

**Table 1:** Overview of the material compositions of transparent neurointerfaces including their relevant properties based on electrical, optical, and mechanical testing

Conductive material	Substrate material	Transmittance (%) @ 550 nm	Avg. site impedance (kOhm) @ 1 kHz	Recording site #	Recording site area (µm^2^)	Mechanical stability without change in impedance	References
ITO	PET	>80	18537	16	196250	-	Kunori^25^
ITO	Parylene HT	>92	369	32	70,685	Up to 100 cycles of bending	Zátonyi^9^
ITO	Polyimide	>80	50.69	32	1,000,000	-	Zátonyi^10^
Au/ Pt	Polyimide	>83	524	32	7850	-	Brosch^107^
Ag nanowire	PET/SU-8	60–90	137.2	9	321,536	Up to 100,000 cycles of bending	Chen^35^
Ag nanowire	PDMS	61.3–80.5	1.3	9	422,500	Up to 500 cycles with 20% strain	Tian^57^
CNT	PDMS	70–90	200	16	10,000	Up to 10,000 cycles with 20% strain	Zhang^30^
Au NW	PDMS	>90	998	16	125,600	Up to 1000 cycles with 20% strain	Renz^33^
Graphene	Polyimide	>80	541	16	2500	-	Kuzum^24^
Graphene	PET	>80	963	16	10,000	-	Thunemann^29^
Graphene	Parylene C	>90	286	16	7854	-	Park^28^
PEDOT:PSS	Payrlene C	N/A	25.8	16	625	-	Donahue^31^
Au nanomesh	SU-8	>65	127,2	N/A	6400	Up to 300 cycles of bending	Seo^51^
PEDOT:PSS	PDMS	>80	50-70	16	90,000	Up to 1000 cycles of bending	Cho^34^
PEDOT:PSS	PEGylated silk fibroin	>85	0.5	4	7,000,000	Up to 900 cycles with 30% strain	Cui^185^
PEDOT:PSS / Graphene	SU-8	84	166	9	900	-	Kshirsagar^186^
Au / PEDOT:PSS nanomesh	Polyimide/SU-8	70-46	30	N/A	6400	-	Qiang^32^
Au/IrOx nanomesh	Polyimide/SU-8	74-62	30	N/A	6400	-	Qiang^32^
Au/IrOx	Thiol/ene-acrylate	>90	6.4	31	10,381	-	Szabó^38^

### Photoartifacts

Depending on the targeted optical stimulation or imaging application, incoherent or coherent light sources are needed. Incoherent light sources emit photons in a randomly oriented phase, contrary to coherent light sources (e.g., lasers), which emit photons in a narrow frequency band with a correlated phase. When recording sites are exposed to a particular material-specific wavelength of light, electrons are excited, and an electric artifact appears in the detected current or voltage signal. This effect is also called the Becquerel effect^45^. Using an incoherent light source, a threshold for the energy of the absorbed photons can be determined as in Eqs. 3 & 4.

3 $$E = hv = \frac{{hc}}{\lambda }$$

4 $$\Psi = hv_0 = \frac{{hc}}{{\lambda _0}}$$

where E is the energy of the absorbed photon, v is the frequency of light, h is Planck’s constant, c is the speed of light, λ is the wavelength of light, and ψ is the photoelectric work function. The value λ_0_ represents the threshold wavelength at which a free charge carrier is generated. The work function of a material can be precisely measured with Kelvin-probe microscopy.

Photoelectric current is induced when the energy of the incident photons exceeds the photoelectric work function. In the case of metallic conductors, the generated current is proportional to the intensity of light and the exposed surface area of the electrode. The location of the focal plane with respect to the electrode material is, therefore, important, as it determines the severity of electrical artifacts in the recordings. For instance, in such a multimodal imaging scheme, excitation of fluorophores during two-photon calcium imaging at a sufficient distance from the transparent electrodes can be completely safe without influencing the quality of electrophysiology recordings^9^.

It should be noted that when multiple photons collide simultaneously (i.e., when using coherent light in confocal or two-photon microscopy), the threshold for photoelectric events scales down nonlinearly. Therefore, multiphoton exposure or optogenetic stimulation leads to the generation of photoelectric artifacts, even though the threshold wavelength necessary to exceed the photoelectric work function in single-photon cases is not reached. A reasonable approach for mitigating the Becquerel effect is the use of conductive materials with a wide bandgap, which requires a compromise during the selection of potential conductive materials. Although liquid ionic conductors (e.g., patch-clamp electrodes) are inherently not sensitive to photoelectric artifacts, they are unsuitable for forming high-density recording arrays. Metal oxides and conducting polymers have been proposed for fabricating transparent electrodes that are less sensitive to direct illumination^46^.

Fluorescence is also a type of photoartifact that occurs when incident light generates lower-energy photons upon collision with the material. This property is also called autofluorescence. In confocal fluorescence microscopy, the sensitivity of detecting changes in signal intensities may be inherently influenced by the implant material. High-autofluorescence materials contribute to high background noise, which can mask slight changes in the signals of fluorophores. Lu et al.^47^ compared the autofluorescence intensities of several polymers used for biomedical applications. They found that polyimide has significantly larger autofluorescence in the relevant biological optical window compared to Parylene C or polydimethylsiloxane (PDMS), while Parylene HT performed even better. The low autofluorescence of Parylene HT was first applied in transparent ECoGs used for two-photon imaging by ref. ^9^.

### Elasticity and stretchability

Compressive and tensile forces inherently act on neural interfaces either during the surgical procedure of implantation or due to micromotions inside the skull. The latter is inevitable, as the brain is floating in cerebrospinal fluid, and heartbeat and breathing lead to periodic mechanical impacts on the tissue through this indirect pathway^48^. It was also shown that during the acceleration of the head, such as in the case of traumatic brain injury, the brain could exhibit 5% strain^49^. Whether the sensor part of the implant is tethered to the skull or not, these phenomena induce cyclic stress in the material, which may also generate shear forces on the material/tissue interface. To alleviate the consequences of such mechanical processes, the elasticity and stretchability of the implant material are of key importance.

If compressive or tensile forces are acting on a solid material, it can respond to the stress on the unit area with strain. Changes in the geometry of the material are reversible within the proportional section of the stress‒strain curve. This proportional elastic deformation can be characterized by Young’s modulus, which can be derived from Hooke’s law. The Young’s modulus of a material equals the compressive or tensile stress exhibited upon the strain of the material (see Eq. 5).

5 $$\sigma = {{{\mathrm{E}}}} \ast \varepsilon$$

where σ is the stress exerted on the material, E is Young’s modulus, and ε is the deformation (or strain) along the direction of the compressive or tensile force.

A low Young’s modulus of substrate materials is favored since the immune response has been found to be modulated by shear forces on the tissue/device interface induced by micromotions inside the skull^50^. In addition to the mitigation of the neuroinflammatory response, the occurrence of strain may also be disadvantageous to the integrity of the layer structure of the interface^3^. Stretchability is expressed as the maximum strain upon tensile forces, which the material can withstand without device failure. Strain along a single axis can be expressed by Eq. 6:

6 $$\varepsilon = \frac{{\Delta l}}{l}$$

where ∆l represents the change in the length of the specimen.

It should be noted that the gradual formation of microcracks can still occur along the direction of a cyclic load at lower strain values. For this reason, some groups have already tested the resistance of several pairs of conductive and substrate materials to periodic elongation and bending^9,33,51,52^. The Young’s modulus and stretchability of some typical substrate materials are shown in Table 2. Even though a soft microdevice can be carefully implanted in such a way that no significant strain acts on the substrate, the conductive layers may show signs of deterioration in signal quality compared to their original states after being micromachined. Therefore, the increase in impedance of the integrated recording sites upon strain should be investigated. For instance, Zhang and colleagues showed that even a 50% change in impedance with respect to the original value could be obtained after electrodes were exposed to 20% strain for several thousand cycles^30^. In addition to reducing the intrinsic stress of the conductive thin film materials, maintaining symmetry in material thickness and maintaining the conductive layer in the neutral axis of the device can also improve resistance to device failure. In this way, even brittle materials, such as indium-tin-oxide, can be embedded and endure many bending cycles^9,10^.

**Table 2:** Relevant physical and technological features of polymer substrates typically used to form transparent neurointerfaces

Polymer materials		Polyimide	Parylene		PDMS	SU-8	PET	Polytetrafluoroethylene (PTFE)	Poly(methyl methacrylate) (PMMA)	Liquid Crystal Polymer (LCP)
Type/Manufacturer		PI 2611 (HD microsystems)	Pary C (SCS)	Pary HT (SCS)	Dow Corning® 184 Silicone Elastomer	MicroChem SU-8 3000	CS Hyde Company 48-F-OC Clear			Dyconex
**Chemical properties**	Moisture absorption (%)	0.5	<0.1	<0.01	0.5	0.55	0.5	<0.01%	0.3–0.4	0.04
	Density (g/cm^3^)	1.082–1.400	1.289	1.32	1.03	1.075–1.153	1.39	2.150–2.200	1.170–1.200	1.4–2.0
**Thermal properties**	Glass transition temperature (°C)	360	80–100	n/a	n/a	200	n/a	127 (rubbery @ RT)	45, 105 (rigid @ RT)	>280
	Thermal conductivity @ 25°C (W/(m*K)	0.105	0.084	0.096	0.2	0.2	n/a	0.23–0.25	0.17–0.25	0.2
	Continuos service temperature (°C)	n/a	80	350	200	300	n/a	n/a	n/a	320
	Lin. coeff. of thermal exp @ 25°C (ppm)	3	35	36	310	52	1.7	100–216	50–90	3–50
	Melting temperature (°C)	n/a	290	>500	226–232	n/a	254	327	n/a	275–330
	Thermal stability @ 5% wt.loss (°C)	620	100	450	200	300	n/a	n/a	n/a	320
**Mechanical properties**	Tensile strength (MPa)	350	68.95	51.71	2.24–6.2	73	193–234	20–35	48–76	52.8–185
	Young’s modulus (GPa)	8.5	2.76	2.55	(0.36) 1.32-2.97	2	4.9–5.1	0.41–1.2	1.8–3.2	8.5–17.2 (40.0)
	Elongation at break (%)	100	200	200	100	4.8	92–115	200–550	2.0–10.0	1.2–7.0
**Electrical properties**	Dielectric Strength (V/mil)	5080	5600	5400	540	n/a	n/a			n/a
	Bulk/Volume resistivity (Ωcm)	>10^6^	8.8 × 10^16^	2.0 × 10^17^	2.9 × 10^14^	7.8 × 10^14^	16 × 10^15^			
**Optical properties**	Transmittance (%)	≥80% @ 520–720 nm	>90% @ 550 nm							
**Biocompatibility**	Classification	USP class VI (eg. Protomide®)	USP class VI	USP class VI	biocompatible (ISO 10993-5, ISO 10993-6)	USP class VI				
**Microfabrication**	Compatible with microfabrication processes of impatble neural prostheses	+++	+++	+	+++	++				

## Transparent conductive materials

As detailed in Chapter Conductivity, a range of inorganic and organic materials are potentially available to form transparent conductive lines and sites that are able to detect and transmit changes in biopotentials in living tissue. In this chapter, these materials are presented in more detail to deepen the understanding of their benefits and limitations.

### Inorganic materials

#### Metal oxides

Transparent conductive oxides are wide-band-gap semiconductors typically doped to attain low resistivity. Among metal oxides, indium-tin-oxide (ITO) and zinc oxide (ZnO) are popular materials for flat panel displays and thin film solar cells in commercially established technology and have also been proposed for use in transparent implants^9,10,53^. In general, increasing the thickness of the metal oxide layer increases conductivity and decreases transparency. The widely used indium-tin oxide is formed by doping In_2_O_3_ with Sn impurities. Oxidation of these impurities contributes to the release of electrons in the conduction band, which leads to a net increase in charge carrier density and, therefore, conductivity. An alternative to ITO is ZnO, which is also one of the few nontoxic transparent metal oxides, and when doped with Ga and Al, a charge carrier density and sheet resistance similar to those of ITO can be attained^54^.

#### Metal nanowires

Metal nanowires are 1D single- or polycrystalline forms of metals. These percolating networks have high flexibility, high optical transparency, and high electrical conductivity^55^. Their high-aspect ratio nature, compatibility with solution-based processing, and large-area deposition techniques make them promising substitutes for brittle conductive oxides. Their resistivity and transparency are determined by the diameter of the individual nanowire, which has a thickness similar to that of metal oxide conductors. Small diameters lead to less scattering of photons, which is favorable for reaching higher transmittance, but a small diameter adversely affects resistivity as it approaches the mean free path length due to the increased surface scattering of electrons^56^. Silver nanowires showing outstanding performance under cyclic strain have only been recently applied in flexible neural implants^57,35^, and they are expected to further increase the mechanical robustness of transparent devices as well.

#### Nanomeshes

Nanomeshes are interconnected networks of metal nanowires. They exhibit low junction resistance and, therefore, high electric conductivity and high transparency. They show only a slight increase in electrical resistance upon strains greater than 100%^58^. Their topology and diameter can be tailored by advanced nanomachining processes to tailor transmittance and conductivity. Gold nanomeshes have been proposed for the formation of transparent electrode layers on flexible substrates for neural applications^51,32^.

### Organic materials

#### Carbon nanotubes

Carbon nanotubes (CNTs) are an allotrope of graphene that exhibit excellent conductivity and tensile strength. The reason behind the negligible sensitivity to light-induced artifacts of carbon nanotubes lies in their band structure, which has multiple Van Hove singularities. Unlike that of metals with a continuous density of states, the photoexcitation of CNTs is influenced by the wavelength of light since absorption only takes place when the photon energy is resonant with the electronic transition energy between mirror image peaks in the density of states^59^. Since there are CNTs with diverse diameters and chirality values in an ensemble, the probability of responding with an excited state is very low. Single-walled carbon nanotubes with a diameter in the nanometer range have been proposed to form transparent conductive layers in neural interfaces^30^.

#### Graphene

Graphene is an emerging 2D material consisting of a single layer of carbon atoms arranged in a hexagonal structure. It exhibits excellent mechanical properties, high electrical and thermal conductivity, and outstanding optical transparency. Despite these beneficial features, current limitations in large-scale production and technology still hinder the application of this material. In principle, a single layer of graphene could be up to 97.7% transparent; however, attaining stability and processability with a monolayer form is challenging^24^. Future improvements in micro- and nanomachining technology will further facilitate the stable application of graphene in flexible neuroelectronic devices^28,29^.

#### Conductive polymers

The easy integration of conductive polymer formation in semiconductor technology makes this group of materials a promising candidate for replacing metal oxides^60^. In addition to their tunable conductivity, they exhibit excellent mechanical properties and stability due to their soft nature. Conductive polymers are composed of conjugated chain structures, which must be doped to reach a conductive state dominantly governed by a hopping transport mechanism^61^. Their usability depends on the stability of the as-created pi-electron system, and synthesizing the optimal composition has been a great challenge. Poly(3,4-ethylenedioxythiophene) (PEDOT) and its derivatives and analogs are some of the most often used conducting polymers^62,63^. Poly(3,4-ethylenedioxythiophene):poly(styrenesulfonate) (PEDOT:PSS) prepared by chemical polymerization in solution is the most popular form of this material, as it can be dispersed in water and some polar organic solvents. The conductivity of PEDOT:PSS has recently reached that of ITO and could be further increased by tuning the molecular weight of PEDOT. A prime advantage of PEDOT is that its surface properties, such as wettability, can also be tailored, which provides further opportunity for surface functionalization with various biological molecules^31,64^.

## Transparent substrate materials

There are various polymers that enable the encapsulation of transparent conductive traces in flexible neurointerfaces. However, their material properties (see Table 2) are of key importance in determining their final application, and their processability and manufacturing schemes also have limitations. This chapter highlights the most common substrate materials used to create transparent neural interfaces. For an overview of already proposed transparent neurointerfaces, see Fig. 2.

### Parylene

The trade name for poly(para-xylylene) is Parylene, which is an outstanding material for multimodal imaging neurointerfaces. It is a thermoplastic polymer material used as a structural material (substrate and encapsulation) and dielectric material for MEMS-fabricated μECoG devices. The monomer structure consists of a para-benzenediyl ring connected by methylene bridges^65^. The chemical vapor-deposited layer is conformal and chemically inert and exhibits low moisture and vapor permeability and a pinhole-free surface due to the room-temperature process^66^. The same name is used for derivatives of Parylene, where hydrogen atoms on the phenyl ring or on the aliphatic bridge are replaced by other functional groups; consequently, they all have distinct chemical compositions and physical properties: Parylene N ([poly(para-xylylene]), Parylene D (poly[dichloro-p-xylylene]), Parylene C (poly[chloro-p-xylylene]) and Parylene HT (poly[α,α,α′,α′‐tetrafluoro‐p‐xylylene]). For Parylene C, the dimer is substituted with one chlorine atom per repeating unit on the aromatic ring, while Parylene HT has a fluorine atom in place of the alpha hydrogen of the N-type dimer. Parylene C is the most common derivative used in various biomedical fields, and its biocompatibility has already been well demonstrated^67–70^. There are several catheters, stents, cochlear implant pacemakers, etc., that have Parylene C-coated components and are already approved by the Food and Drug Administration Agency (FDA). One disadvantage that hinders the exclusive application of Parylene C as a structural material is that the polymer tends to delaminate during long-term experiments^71,72^. Parylene HT has a continuous service temperature of up to 350 °C, while polymers either from the Parylene family or others used for neural applications tolerate only lower process temperatures. In addition to its superior thermal characteristics, Parylene HT has the lowest dielectric constant among Parylene alternatives (SCS, Data Sheet). Parylene HT exhibits low initial autofluorescence with higher UV light stability, and its transmittance is also superior to that of other polymer materials^47^. Regarding the transmittance of Parylene HT, values greater than 90% can be measured between the biologically relevant wavelength range of 400–980 nm, which is significantly higher than that of polyimide (PI)^10^ and polyethylene terephthalate (PET)^73^ and similar to that of SU-8 substrates^74^ and polydimethylsiloxane (PDMS)^75^. The lower autofluorescence of Parylene HT films is associated with the presence and strength of the C-F bond. Under UV excitation, photooxidation, and fluorine extraction are also unlikely to occur. In the case of Parylene C, long-time UV excitation induces lower autofluorescence; however, it also negatively affects the flexibility and transparency of the polymer. Based on the observation of B. Lu et al., Parylene C turns brittle and yellow after long-time UV exposure due to the formation of C=C bonds and carboxyl groups during the photooxidation process. If a measurement is sensitive to background fluorescence, for instance, multiphoton microscopy and fluorescence detection, Parylene HT is the best candidate due to its moderate autofluorescence profile under UV illumination^47^. UV light stability is also beneficial during microfabrication, where the polymer is patterned by photolithography. In the construction of μECoGs, Parylene can be patterned using reactive ion etching techniques.

There are several recently presented neural microimplants that exploit the beneficial properties of the Parylene family. Neto, J.P. et al. reported a Parylene C/silver nanowire (Ag NW)/indium zinc oxide (IZO)/Parylene C electrocorticography electrode array for neural recording^36^. As the adhesion between the Ag NWs and Parylene C is inherently weak, the IZO coating was used to improve Ag NW adhesion to the substrate, which was validated through a Scotch tape test. They also validated the operation of their device under fluorescence microscopy by imaging mouse brain slices and by recording electrical activity from the surface of the rat cortex under anesthesia. Ledochowitsch, P. et al. presented an approach for combining microelectrocorticography with optogenetics. To minimize photoelectric artifacts, they used indium-tin oxide (ITO) to form the conductive components and Parylene C as the substrate and encapsulation material^21^. They showed the feasibility of the multilayer device by demonstrating simultaneous optical stimulation and electrophysiological recording in the rat cortex at three weeks postimplantation. Zátonyi et al. reported an implantable μECoG with a transparent indium-tin-oxide conductive layer and Parylene HT as the structural material^9^. They showed that Parylene HT layers did not delaminate even after soaking the final device for 24 days in saline and also proved the electrochemical stability of ITO recording sites. During in vivo tests, fluorescent signals of labeled neurons recorded in brain slices were visible without significant distortion in the dimensions of the neuronal structures. The implants were successfully tested in simultaneous two-photon Ca^2+^ imaging and cortical electrophysiology experiments. Fluorescent signals of individual neurons were recorded at a sufficient signal-to-noise ratio even after 51 days in a chronic experiment. Kwon, K. et al. reported a Parylene C/ITO/Parylene C electrocorticography array with embedded light-emitting diodes (LEDs) for optical neural stimulation of channelrhodopsin-2 (ChR2) in genetically modified species^20^. Donahue, M. J and Kaszás, A. et al. demonstrated a method for the combination of fluorescence signal collection through two-photon imaging and electrophysiological recordings with PEDOT:PSS conductive polymer electrodes^31^. They were able to record seizure events and the corresponding optical signals from the cortical surface of transgenic Thy1-GCaMP6f mice with neurons expressing a fluorescent calcium indicator. Dijk, G. et al. reported a novel method for the microfabrication of transparent devices with PEDOT:PSS electrode pads and interconnects on a PaC substrate, tested in mice with two-photon imaging^76^. Takei, A. et al. reported a Parylene C/PEDOT:PSS/Parylene C/PDMS test structure and tested its stretchability and durability^64^. The PEDOT:PSS layer was conductive even when stretched up to 170%, and the change in resistance was less than 10% after 4000 cyclic loads. Susloparova, A. et al. reported the microfabrication and characterization of a transparent PEDOT:PSS/Parylene C microelectrode array (MEA) on glass slides^77^. They validated their MEA by recording the spontaneous neural activity of primary cortical neurons cultured over 4 weeks on PEDOT:PSS electrodes. Park, D.-W. et al. developed transparent Parylene C/graphene/Parylene C µECoG arrays and tested their performance in vivo by implanting them on the cerebral cortex of a Thy1:ChR2 mouse for optogenetic stimulation concurrently performed during the recording of neural activity with graphene electrode sites^8^. Later, they tested a graphene-based carbon-layered electrode array (CLEAR) in electrical stimulation experiments wherein the stimulation pulses were delivered to the cortex through a graphene electrode site and the corresponding neural activity was visualized via fluorescence in GCaMP6f mice^28^. A Parylene C/graphene-based microelectrode array was also successfully utilized to perform cortical electrophysiology in optical coherence tomography (OCT) experiments^78^.

### SU-8

SU-8 is an acid-catalyzed, epoxy-based negative photoresist composed of bisphenol-A novolac epoxy resin (EPON® SU-8 resin), organic solvents such as cyclopentanone or GBL (gamma-butyrolactone), and a 10% weight photoacid generator, such as triarylsulfonium hexafluoroantimonate salt. Since it was introduced by IBM in the late 1980s, SU-8 has gained significant attention because it is compatible with conventional microfabrication processes, such as photolithography, spin-coating, and dry etching techniques, and suitable for forming high-aspect-ratio microstructures^79,80^. SU-8 absorbs UV light, which is followed by a decomposition step of the photoacid generator, which protonates the epoxy groups. Polymerization is completed only during the postexposure bake, when the temperature is raised^52^. The fully cured polymer film has an exceptionally high degree of cross-linking due to its low molecular weight (~7000 ± 1000 Da) and the presence of epoxy functional groups^81^. As a result of the highly cross-linked polymer matrix, SU-8 has excellent mechanical properties, such as its relatively low Young’s modulus (~2–3 GPa), thermal stability (~300 °C), and high tensile strength (73 MPa, only polyimide has a higher tensile strength). In addition to beneficial mechanical properties, SU-8 has further advantages in MEMS fabrication, such as cost-effectiveness, tailorable film thickness in multiple coating steps^82^, and high transparency in the biologically relevant wavelength window (400–980 nm). SU-8 has already been applied in various in vitro and in vivo electrophysiological experiments^83,84^.

Regarding neural applications, SU-8 is mostly combined with other substrate materials to form a multilayer stack, such as PDMS/SU-8^30^, polyimide (Kapton)/SU-8^32^, Parylene C/SU-8^85^, or PET/SU-8^29^. The reasoning behind this approach lies in the extremely low value of fracture strain (SU-8 can only withstand 4.8% elongation before crack formation); therefore, the other polymer layers act as a flexible support for SU-8. Further difficulties are introduced by the application of the hybrid layer-stack, as this may lead to the curling of the final device when peeling them off from the handle wafer (usually silicon or glass). Additional issues arise during the manufacturing process. As described before, the processing of SU-8 is similar to that of other negative photoresists; however, in the postbaking step, time and temperature need to be carefully considered. Increased baking time or temperature induces stress formation in thicker layers, resulting in crack formation during solvent evaporation. These cracks can cause unwanted lift-off at the developing step and weaken the advantageous mechanical properties of the final product^81^. Recent publications have demonstrated that antimony leaching from the final, cross-linked product is minimal and cytocompatible. In 2002, Kotzar, G. et al. performed in vitro physicochemical and cytotoxicity tests and in vivo cytotoxicity studies to evaluate the local tissue response following ISO 10993-5 and ISO 10993-6 standards^86^. Their results indicated that the amount of cytotoxic extractable substances in the aqueous and nonaqueous media was below the recommended amount (less than Grade 2, which indicates mild reactivity). Márton, G. et al. provided a comprehensive analysis by implanting SU-8 3000 penetrating microprobes chronically in the neocortex of 21 rats for 8 weeks. Their immunohistochemical results indicated that the biocompatibility of the SU-8 material is high^87^.

The versatile application of SU/8 in microimplant structures used for concurrent electrophysiology and two-photon Ca^2+^ imaging was recently demonstrated by ref. ^85^. They developed polyimide (Kapton)/gold/PEDOT:PSS nanomesh/SU-8- and polyimide/gold/IrOx nanomesh/SU-8-based electrode arrays^32^ and captured GCaMP6s protein activity in the neurons in layer 2/3 of the mouse visual cortex.

### Polyimide

Polyimide products are supplied as polyamic acid precursors synthesized in a two-step process from aromatic tetracarboxylic dianhydrides and aromatic diamines. To form a spin-coatable liquid solution, a dipolar aprotic solvent is needed, such as an n-methyl-2-pyrrolidone (NMP)- or n,n-dimethylacetamide (DMAc)-based solvent. The chemical structures of diamine and dianhydride have an extensive effect on the physical and chemical properties of the final product. Recently, manufacturers switched from pyromellitic dianhydride (PMDA) and 4,4’-oxydianiline (ODA) to biphenyltetracarboxylic dianhydride (BPDA) and paraphenylenediamine (PPD) with higher hydrolytic stability and lower water uptake, yielding polyimides for long-term implantation^88^. In 2010, Rubehn and Stielglitz researched the long-term stability of BPDA-PPD polyimides (PI 2611 – HD Microsystems, U-Varnish-S – UBE, Durimide 7510 – Fujifilm (type: information not provided)). They concluded that after 20 months in phosphate-buffered saline solution at 37 °C to simulate body temperature and at an elevated temperature of 60 °C to accelerate aging, no changes in material behavior could be observed by analyzing the monthly recorded stress‒strain curves and determining mass loss^89^. Polyimide materials are also characterized by extremely high thermal stability and glass transition temperature (~360 °C), which makes them suitable for most thin film metallization processes. Due to their coefficient of thermal expansion being similar to that of silicon, polyimides have become an attractive substrate and encapsulating dielectric material for implantable devices (e.g., retinal prostheses^90^). The thickness of the polyimide layer can be varied by varying the spin speed in a clean-room environment with a relative humidity level of less than 50% to avoid unwanted delamination of the polyimide film. Fully polymerized thin films are flexible and show excellent mechanical properties. Polyimides have a high Young’s modulus (~8.5 GPa) and tensile strength (~350 MPa). Although thin film polyimides are flexible, their stretchability is limited (100% elongation before fracture formation). Their other disadvantageous property is their limited transparency compared to that of other popular implant materials (e.g., Parylene). Dry etching (deep reactive ion etching or reactive ion etching) is the most common technique for patterning polyimide materials.

In spite of the limited optical transparency and high autofluorescence of polyimides, several neuroimaging applications with PI microstructures have been demonstrated. Zátonyi, A. et al. presented the fabrication, characterization, and in vivo application of a polyimide-based μECoG array^10^. In their work, they introduced a micromachined polyimide/ITO device, which facilitated the parallel detection of ECoG signals and intrinsic optical signal imaging in the cat’s primary visual cortex. They concluded that tolerance to bending loads could be improved by forming an indium-tin oxide layer in the neutral axis of the thin film stack, which is especially complicated, e.g., in the case of ITO-coated PET substrates. They validated the in vivo performance of the arrays by reconstructing functional domains through transparent arrays under 609 nm illumination of the exposed cortical surface. This was the first in vivo demonstration of a flexible, polyimide/ITO/polyimide-based μECoG array that was used for the simultaneous detection of intrinsic optical signals in conjunction with cortical EEG. Lu et al.^26^ and Kuzum, D. et al.^24^ developed and tested polyimide/graphene μECoG arrays. Kuzum et al. presented the use of transparent arrays during calcium imaging in hippocampal brain slices and recorded high-frequency activity, which cannot be observed by slow calcium transients in calcium imaging. They fabricated graphene microelectrodes on flexible 25-μm-thick polyimide (Kapton, DuPont) substrates. Lu et al. have demonstrated the feasibility of graphene microelectrode arrays in high-precision stimulation experiments on the barrel cortex in an adult rat model. Porous graphene spots were first patterned on 50-μm-thick Kapton using direct laser pyrolysis.

### Polydimethylsiloxane

Polydimethylsiloxane (PDMS) is the most common silicone material with a backbone made of siloxane functional groups substituted by methyl side groups attached to silicon (Si) via a single bond -[R1R2SiO]n-, where n is the number of repeating units^91^. Since methyl is a reactive side group, a variety of other functional groups can be attached to it; therefore, polymers with diverse physical and chemical characteristics can be prepared. Reactive side groups are beneficial for adhesion as well^92^. Polysiloxanes are extensively used in biomedical applications, such as effective long-term encapsulation of implantable neural prostheses^18^, cochlear implants^93^, cuff electrodes^94^, bladder^95^, and bowel controllers. They have gained popularity due to their elastomeric properties, such as flexibility and extremely low elastic modulus (~1–3 × 10^−3^ GPa) compared to that of other materials, such as Parylene, PI, SU-8, and PET. The foreign body response to PDMS after implantation is minimal, and in terms of biocompatibility, it is classified as a USP class VI material^18^. Despite these beneficial properties, there are a few reasons that make PDMS applications challenging. Swelling, poor adhesion between two layers (polymer–polymer, polymer–dielectric material) due to contamination between the surfaces, and inaccurate control of process parameters during spin-coating and curing, leading to the entrapment of air can all result in the delamination of conductive metal layers^91^. The void-free deposition of PDMS layers is critical because liquids can penetrate through the material. Its thermal expansion coefficient is different from that of metals, causing adhesion issues during the microfabrication processes. When oxygen plasma activation is needed (e.g., to add silanol groups (SiOH) to the surface or to properly clean the surface), weak plasma power and short plasma time are needed (below 50 W) to avoid crack formation in the structure of the cured polymer. However, the integration of PDMS into the microfabrication process flow is straightforward, although the aforementioned aspects need to be carefully considered in the design phase.

Some recent papers have reported examples of PDMS-based transparent implants. Tian, J. et al. reported on a stretchable, transparent PDMS microelectrode with silver nanowires (Ag NWs) and tested its optical, electrical, and mechanical properties before the electrophysiological recording and optogenetic pacing of mouse hearts^57^. PDMS (Sylgard 184, Dow Corning, Midland, MI, USA) was used as a substrate and encapsulation layer. Zhang, J. et al. demonstrated a PDMS/SU-8-based μECoG array with a carbon nanotube (CNT) thin film and its performance in simultaneous electrophysiology and two-photon calcium imaging experiments on the cortical surfaces of GCaMP-expressing mice^30^. Their results indicated that with this combination of insulating and conductive materials, the real-time, continuous electrophysiological monitoring of cortical activity under mechanically active conditions (e.g., traumatic brain injury) is feasible. Wang et al. demonstrated a hydrogel-elastomer neural interface with artificial cerebrospinal fluid (ACSF)/polyvinyl alcohol (PVA) hydrogel as a conductive material and PDMS as a dielectric shell^37^.

### Polyethylene terephthalate

Polyethylene terephthalate (PET) is a thermoplastic polymer produced via polycondensation between terephthalic acid (TPA) and ethylene glycol. PET building blocks are the polymerized units of the monomer ethylene terephthalate, with -[C_10_H_8_O_4_]-_n_ repeating units. Depending on its synthesis, PET may exist as both an amorphous and semicrystalline polymer. The transparency of the final product varies depending on the particle size and the degree of crystallinity^96^. PET has a partially aliphatic and aromatic structure and is a polyester polymer^97^. Its commercial importance is undisputable due to its application in nonbiomedical (e.g., synthetic fibers for textiles, packaging, automobile industry, etc.) and biomedical fields (e.g., bone regeneration, synthetic bypass graft for the replacement of large diameter (>6 mm) blood vessels, surgical suture membrane, angioplasty balloon, support in breast implants, scaffold for tissue engineering, ligament prosthesis, etc.)^96,98^. In the biomedical field, PET is advantageous as a chemically inert, biocompatible material with high hydrolytic stability due to the presence of aromatic rings^99^. Its Young’s modulus and tensile strength are between those of polyimide and Parylene (~5 GPa and ~200 MPa, respectively). PET is compatible with most standard microfabrication processes due to its coefficient of thermal expansion being similar to those of polyimide and silicon at 25 °C (~2 ppm). However, in the fabrication of neural prostheses, PET is always applied as a substrate in the form of commercially available sheets, which makes it challenging to embed the conductive components in the neutral plane of the device.

Chen, Z. et al. demonstrated a flexible, transparent silver nanowire (Ag NW) microelectrode array (MEA), where a 25 μm thick PET film (CS Hyde Company) was the substrate and a 7-μm-thick SU-8 layer was the encapsulation material^35^. They successfully validated the device performance using Langendorff-perfused mouse and rat hearts during cardiac dysfunction and demonstrated that the Ag NW MEAs enabled the real-time monitoring of heart rhythm during optogenetic pacing and optical mapping. Kunori, N. et al. presented the feasibility of an indium-tin oxide (ITO)-coated PET sheet-based, epidural cortical array for electrophysiological stimulation in conjunction with voltage-sensitive dye (VSD) and optical imaging^25^. They emphasized that their fabrication method is simple and cost-effective. The in vivo performance was demonstrated by placing the device epidurally over the sensorimotor cortex of adult Wistar rats. Lu, Y. et al.^27^ and Thunemann et al.^29^ presented PET/graphene/SU-8-based μECoG arrays using similar methods. In the work of Thunemann et al., a 50-μm-thick PET film (CS Hyde Company, Mylar 48-02F-OC) was used as the substrate material. During the microfabrication process, 20-μm-thick PDMS as an adhesive layer was used between the silicon wafer and the PET film. Graphene was transferred onto the prepatterned area using bubbling transfer and the PMMA method. They validated the device performance in a simultaneous process of two-photon imaging of neuronal and vascular structures, two-photon calcium imaging, optogenetics (OGs), and local field potential (LFP) recordings of the cortical surface of Thy1-ChR2 transgenic mice expressing the OG actuator channelrhodopsin-2 (ChR2) in layer V pyramidal neurons. Recently, Cho et al. demonstrated the microfabrication method and in vitro and in vivo validation of a PET/PEDOT:PSS/SU-8 μECoG array^34^. They emphasized that the developed technology is low-cost and facile without complex process steps such as the graphene transfer method. The in vivo performance of the proposed material composition was confirmed in a simultaneous process of electrophysiological signal recording of the cerebral cortex of a Thy1-ChR2-YFP mouse and optogenetic stimulations with various laser intensities.

## Strategies to alleviate electrical artifacts

As discussed previously, light can have multiple effects on both conductive elements and substrates, such as the photoconversion effect, the photothermic effect or fluorescence itself^100^. All of these effects also depend on the light used, which can be incoherent light or coherent light, depending on the methods of imaging or stimulation used. Moreover, the wavelength of the light can also differentially affect the recordings, depending on the materials used in the design of the electrodes; e.g., gold and titanium are greatly affected throughout the visible wavelength range up to the infrared wavelengths, while PEDOT:PSS is mostly affected only in the infrared wavelength range above 1400 nm^101,102^. Regarding the wavelength used in techniques, visual wavelengths ranging from ~400 to 700 nm show considerable impact on most opaque conductive materials and substrates. The corresponding techniques include voltage-sensitive dye imaging, brightfield imaging or fluorescent imaging, and optogenetic stimulation, which is usually performed between the 400 and 500 nm regime^19^. The application of conductive materials could potentially make use of this phenomenon, whereby illumination can be used as a wireless stimulation source, as demonstrated with free-floating carbon-fiber electrodes for the stimulation of GCaMP3-positive neurons in the mouse in vivo^100^. In regard to the wavelength range, both incoherent and coherent light can be used for different applications. Incoherent light is mostly used when using voltage-sensitive dye imaging or brightfield imaging, while it can also be used for standard fluorescence microscopy techniques. Coherent light is mostly used in confocal microscopy applications, which can involve both structural and functional imaging. The most common application of coherent laser light in both single-photon and multiphoton imaging is functional calcium imaging, which can be conducted both with injected synthetic dies or using transgenic animal models. The most common animal model used currently is the GCaMP transgenic mouse animal model, with different versions of the genetically encoded calcium indicator (GECI) protein, such as the versions with a slow decay time but high fluorescent yield, the older versions of both GCaMP3 or GCaMP6^103^ that are kinetically fast but have a lower signal-to-noise ratio (SNR) or the newer versions of the indicator, such as GCaMP7^104^ or GCaMP-X^105^. Despite the controversy regarding the imaging of voltage-sensitive proteins incorporated into the membrane of neurons in the brain, recently, there have been significant developments regarding that area, making combined imaging and electrophysiology a feasible choice^44^. Since these applications require electrodes that are compatible with imaging, it is reasonable to microscopically examine the characteristics of electrodes.

One crucial aspect when examining the compatibility of electrodes with microscopy techniques is their transparency and interaction with light. Here, in addition to the abovementioned interactions, the most important interaction is the photoelectric effect. Since light can induce both imaging and electrophysiology artifacts in both modalities^100^, when the recording types are combined, it is necessary to consider the photoelectric artifacts of different materials with regard to different characteristics, such as the transparency, wavelength dependence, or effects of incoherent or coherent light. Moreover, it is important to examine the single-photon versus multiphoton responses of different electrode materials.

**Fig. 3:**
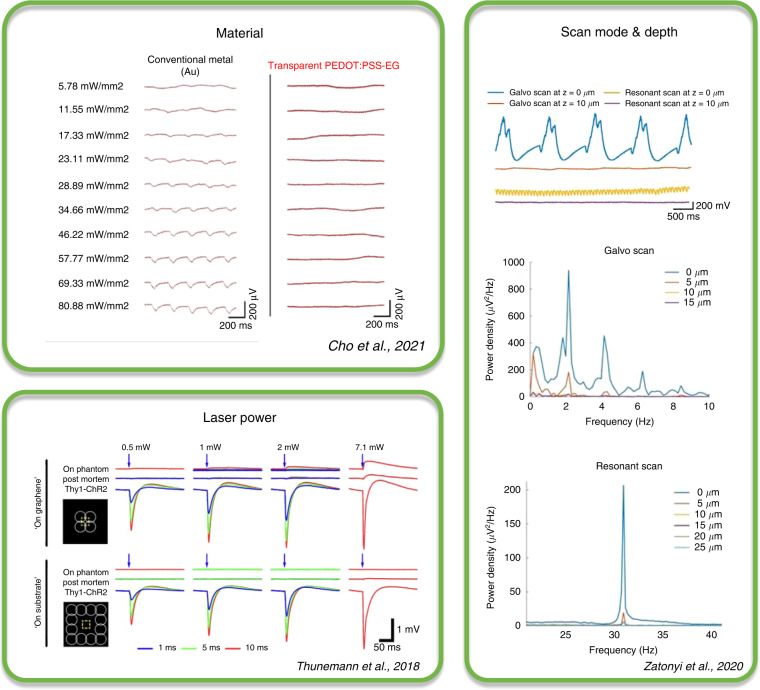
Photoelectric artifacts. Summary of typical experimental conditions that lead to photoelectric artifacts when using transparent micro-ECoG arrays in combination with optical imaging or stimulation methods

As we have shown above in Table 1, multiple materials are used in microfabrication to achieve the multimodality of devices that allow the combined use of optical and electrophysiological methods. In addition to the well-known effects of light-induced photoactivation, such as the photovoltaic effect, photothermal effect, or induced fluorescence in the applied electrical recording device^100^, photoelectric artifacts induced upon light exposure on the conductive layers, introduced in Chapter Photoartifacts, may hinder noise-free electrical recording and limit the evaluation of experimental outcome. The most typical experimental conditions that lead to photoelectric artifacts are shown in Fig. 3. In addition to the electrode material, the laser parameters, scanning mode of the microscopy, and depth of the focal plane may be limiting. Versatile experimental conditions make it difficult to objectively compare measurement results reported for transparent arrays. Furthermore, most of the listed studies focus on specific imaging methods combined with electrical measurements or stimulation, meaning that there are wavelength-specific requirements for the conducting materials used. Therefore, we examine the feasibility of different materials by examining the wavelength ranges common for specific imaging methods.

### Incoherent light-induced artifacts

The first modality for imaging is the visible wavelength range (from 400 to 700 nm), which is suitable for standard brightfield microscopy and fluorescence microscopy methods or optical stimulation using optogenetics. Transparent devices in this regime can be used for the identification of anatomical regions through structural dyes^106^. Regarding functional imaging with incoherent light sources, voltage-sensitive dye imaging (VSDI) provides a means for increased spatial resolution when combined with transparent devices. The technique has been applied in different preparations, such as the mouse heart^35^ or the rat visual cortex^107^. Although constant illumination is used here while recording electrophysiology, the reports show no artifacts caused by light, possibly because of the use of low light intensities to avoid the well-known bleaching and phototoxic effects of voltage-sensitive dyes. Using VSDI through micro-ECoG devices allowed the recording of cortical stimulation events, such as whisker stimulation^25^ or optogenetic stimulation^19^.

Another way to track functional neuronal activity is calcium imaging, where neurons express genetically encoded calcium indicators (GECIs)^108^ or are injected with synthetic calcium indicator dyes, such as Fluo4-AM or OGB-1AM^109^. Since neuronal activity is accompanied by elevated levels of intracellular calcium, these indicators signal these increases through changes in their fluorescence. When used in conjunction with local LFP recordings, calcium imaging can easily provide the much-needed spatial map for completing the high temporal resolution maps of ECoG devices. Widefield calcium imaging combined with local LFP recordings has been used to monitor the evolution of epileptic seizures and provided an impressive method for following the trajectory of seizure evolution over time^42^. It has also been used in conjunction with ECoG recordings in the mouse somatosensory cortex to follow the stimulation of different modalities^33^. Interestingly, papers do not report having electrical artifacts when using widefield calcium imaging combined with ECoG probes, possibly because of the low light intensity levels that are insufficient for the work function of conductive materials used.

### Optogenetics and light-induced artifacts

When light is used to evoke activity either through genetically encoded light-activated proteins^110^ or infrared neuronal stimulation (refs. ^9,106^), the stimulation light pulses can also induce artifacts in the transparent devices, just as during recordings. Direct illumination of electrode sites with high light intensities (>5 mW/cm^2^) produces significant artifacts on metal lines composed of chromium, gold, and platinum^107^. The effect has been measurably different for chromium, gold, titanium, platinum, and iridium, with iridium deemed the best choice regarding light-induced artifacts^111^. On the other hand, in a different study, platinum sites fabricated on a PaC substrate showed a negligible Becquerel effect under in vivo conditions in mice^19^. In general, light-induced artifacts should be measured, and their elimination should be studied using glass electrodes since they have no photoelectric effect^24,112^.

### Coherent light-induced artifacts

Coherent light applications are used for scanning microscopy techniques where the laser light is scanned through the tissues with various methods. As opposed to incoherent light, only scattering affects the number of photons that reach the sample at different distances since the light beam does not diverge with distance^100^. Naturally, scattering can arise both from the sample under scrutiny and from any scattering material placed in the beam path, such as recording or stimulation devices. The choice of materials for these devices used greatly determines whether the transparent device is applicable for imaging or optical stimulation using optogenetics. Gold as a conductive material has been shown to be prone to artifacts when compared with carbon nanotubes (CNTs) in the brains of mice^30^. When using multiphoton imaging combined with electric recordings, the two-photon effect that allows for deeper tissue penetration using infrared light can also be a source of problems. Since the two-photon effect can only take place when light intensities are quite elevated (approximately a millionfold higher than that of single-photon imaging) for at least a brief amount of time (~100 fs)^113^, the great power comes with a greater chance of Becquerel effects. The common measurements for transparency are usually valid for incoherent light, but the nonlinear effects of coherent light lead to the appearance of photoelectrons. A clear example of this is the laser-induced artifact that is produced when scanning through otherwise transparent materials, causing recurrent electrical noise^9^. Although because of its repetitive nature, the induced noise seems easily subtractable, unfortunately, it is specific to a given field-of-view, since laser scanning rarely crosses the conductive material the same way in a different experimental session—thus making its removal very tedious. The effect is even more prominent with more sophisticated scanning methods, such as holographic scanning or 3D random access scanning.

### Solutions for photoelectric artifacts

Despite the multitude of cases described above where the conducting material is prone to light-induced artifacts, many solutions exist even for the opaquest and most susceptible materials, such as titanium-gold. The most obvious workaround for using simultaneous imaging and electrophysiology is to avoid having any opaque materials in the imaging field-of-view. This can be achieved by designing an imaging window in the device itself, which is a solution that can be used when even the substrate is opaque, such as in liquid crystal devices^114^. Since the photoelectric effect is proportional to the number of photons that reach a given area, a design-based strategy is to decrease the surface area of conductive elements in materials that are prone to photoelectric artifacts. Another solution is to apply materials with morphologies such that light-based limitations are circumvented, although the substrates used would themselves be opaque, such as gold nanomeshes combined with various transparent materials, such as PEDOT:PSS^85^. With transparent devices, the substrate (e.g. PaC, PDMS, PET, SU-8) often does not hinder imaging and has high transmittance values, allowing a wider application range for light-based techniques. In addition to using clever designs, experimenters should use transparent devices that do not constrain the experiment in terms of anatomical areas or imaging modalities. In addition to using transparent substrates, such as PaC or SU-8, transparent interconnects and electrode pads have been fabricated with transparent conductive materials, such as graphene^24,29^, CNTs^30^, ITO^9^, and PEDOT:PSS^40,76^. In multiphoton microscopy, extreme light intensities are only present near the focal point of scanning, and values drop quadratically with distance. Consequently, the best solution besides choosing materials that have transmittance in the 400–1040 nm range for two-photon imaging is distancing the imaging plane from the electrophysiological recording plane. As shown, focusing away even slightly (30 µm) from the device can increase the limits of tolerance considerably, approximately fourfold^9^. Others report keeping a 50 µm distance between the scanned area and the device conducting lines or recording sites to avoid photoelectric issues^33^. Although this seems controversial since the aim is to record the two modalities at the same time and place, it still allows the following of widespread cortical effects, such as epileptic seizures and single-cell activity of deeper cortical layers in the brain^30,31,76^. A possible optical solution would be to design optical scanning patterns that allow the laser to be turned off when the scanning path reaches the electrodes or leads, although this is obviously a partial solution, since it does not allow the complete imaging of biological samples.

## In vivo biocompatibility

As previously mentioned, an ideal device that enables concurrent multimodal neuroimaging should be flexible, durable, and tear-resistant. This preferable highly biocompatible device should elicit no mechanical damage, evoke no immune response in the surrounding tissue, and cause no physical damage due to the micromovement during the experiment.

ECoG devices on the surface of the dura mater (epidural) or under the dura mater (subdural) allow the recording of electrocorticograms of the surface of the cerebral cortex for long durations with moderate damage to the underlying and surrounding tissue environment and cause fewer histological and immunoreactive responses than intracortical microelectrodes (refs. ^115,116,117^). The implantation of ECoG devices is less invasive than that of intracortical devices, although they can maintain an interface with the underlying neural tissue. In addition to stable electrical properties, low impedance, good signal-to-noise ratio, stable performance, and appropriate mechanical properties, they also provide long-term biocompatibility in in vivo applications. Therefore, the biocompatibility of ECoG devices is similarly important for stable electrochemical characteristics and needs to be tested at different timepoints in in vivo experiments.

There are extensive studies on tissue response characterization in the case of deep electrode implantation; however, detailed tissue characterization following surface ECoG measurement is still rare in the literature. ECoG devices have mostly been used in acute in vivo experiments thus far;^24,31^ however, their chronic application has also been published recently (refs. ^38,118^). In addition, other chronic surface devices, such as graphene solution-gated field-effect transistors (g-SGFETs), was demonstrated recently^119^.

In this chapter, we summarize the available histological techniques used for different species and review the recent advances in novel methods that may improve the biocompatibility of transparent ECoG devices.

### Macroscopic tissue changes around ECoG devices—tissue growth and encapsulation

A few studies have investigated the possible encapsulation and tissue outgrowth after ECoG device implantation. Macroscopic fibrotic growth was found in some cases, and encapsulation or dural thickening was reported following brain surgery^120^. In Sprague Dawley rats, 1 week after epidural implantation of a Parylene C-Au/Pt ECoG array, connective tissue overgrowth was observed^22,23^. Dural thickening under the ECoG electrodes was observed until 419 days, whereas glial encapsulation was observed one month after the surgery^23^. Dural thickening 6 months following the implantation of a subdural implant was reported to correlate with an increase in 1 kHz electrical impedance at 1 week, with stabilization at 18 weeks postimplant^121^. Calcification areas extending from the edges of the craniotomy to the center were observed after subdural implantation of a silicone-Pt/Ir-based device^122^, and these areas covered nearly 50% of the surface under the electrode array. No significant vascular differences were observed between brain regions located away from the implantation site, brain regions beneath the control window, and brain regions beneath the epidurally implanted Parylene C-Au/Pt micro-ECoG device^22^.

Device characteristic changes over time have been monitored in the long term in several studies (refs. ^23,37,107,118^^,^^123–125^). The impedance of the epidurally implanted polyimide-encapsulated Au/Pt electrode was increased just after implantation, dropped in the first week until the fifth week of implantation, and then returned to the original value following explantation^107^. Within 20 days following implantation, a general trend of steeply increasing (between 150 and 300 kΩ) then plateauing was observed. This impedance change occurred within the period when histopathological tissue changes were observed in the chronic preparation^22^.

Using conformable or nonconformable epidurally implanted polyimide-based multispecies arrays, an increase in the impedance of the conformable device was shown three weeks following implantation, whereas, in the case of conformable devices, it was stable until the fifth week^123^. The increase in impedance was measured following epidural implantation of the thio-lene/acrylate substrate Au micro-ECoG device on the 6th and 12th days, after which it remained stable until the 61st day and then showed a decrease from the 68th day^118^. The impedance of subdurally implanted SWCNT electrodes slightly changed, whereas that of the Pt electrodes used for comparison in the study increased substantially^124^. More recently, a softening ECoG device composed of a thiol-ene/acrylate substrate and IrOx conductive film showed stable impedance characteristics from two weeks until 10 weeks after implantation^118^.

### Quantification of mechanical trauma, cell loss, and neurodegeneration caused by the mechanical action of ECoG devices

Since ECoG arrays do not penetrate the cerebral cortex, they avoid blood‒brain barrier disruption and cause less mechanical trauma between the relatively stiff ECoG device and the relatively soft surrounding tissue than in the case of intracortical electrode implantation (see review^116^). This minimal invasiveness and potentially flexible character of ECoG devices mitigate cortical foreign body responses. However, the implantation procedure and subsequent micromovement of the device on the surface of the dura mater or on the cerebral cortex can still cause constant mechanical trauma resulting in damage, cell loss, and continuous neuroinflammatory responses. Therefore, long-term chronic biocompatibility evaluation of surface multiarray devices needs to be investigated before further possible translational applications in medical diagnosis or treatment. A few studies have evaluated the long-term host-tissue response to either epidural or subdural ECoG devices. The biocompatibility of different types of ECoG devices was examined in several types of experimental animal species with very different survival times following implantation (Table 3).

**Table 3:** Biocompatibility of transparent neurointerfaces characterized by histological methods

Reference	Substrate material	Conductive material	Location	Recording	Imaging	Long-term electrode characterization In vivo	Model animal	Histological staining	Implant duration
Brosch, ^107^	polyimide	Au/Pt	Epidural	+	+	+	Mongolian gerbils	Nissl, Iba1, GFAP	28 or 31 weeks
Vomero, ^123^	polyimide	GC Pt IrOx PEDOT:PSS	Epidural	+	-	+	Long Evans rats	Nissl (thionin)	6 weeks
Ceyssen, ^136^	polyimide covered with ECM	Pt	Epidural	-	-	-	Wistar rat	NeuN, GFAP	3 and 13 weeks
Schendel, ^23^	Parylene C	Au/Pt	Epidural	-	+	+	Sprague Dawley rats	thickness of the collagen	from 2 to 419 days
								meningeal tissue response	1 month
								H&E, Iba1, GFAP	1 year
Matsuo, ^131^	Parylene C	Au	Epidural	+	-	-	Rhesus macaque	H&E	4 weeks
Fedor, ^118^	thiol-ene/acrylate	Au	Epidural	+	-	+	C57BL6/J mouse	NeuroTrace, DAPI, GFAP	80 days
Vitale, ^142^	Parylene C with ECM-coating	Au	Subdural	+	-	-	Sprague Dawley rats	Iba1, GFAP	1 week 1 month
Schweigmann, ^114^	LCP	Au/nanoPt	Epidural	+	+	-	mouse	DAPI, Iba1, GFAP	3 and 28 days
Pavone, ^124^	SWCNT/MDPE	Pt	Subdural	+	-	+	rats	Nissl, NeuN, Iba1, GFAP	2 months
Wang, ^37^	hydrogel	Ag/PVA	Subdural	+	+	+	cats	Iba1, GFAP	2,3,4 or 14 weeks
Yan, ^132^	silicone	Pt	Subdural	-	-	-	beagle dog	H&E, NeuN, Iba1, GFAP	6 months
Degenhart, ^117^	silicone	Pt	Subdural	+	-	-	Rhesus monkey	Hoescht, NeuN, Iba1, GFAP, Vimentin	666 days
Sauter-Starace, ^122^	silicone	Pt/Ir	Subdural	+	-	-	sheep	Nissl, CD11b, GFAP	10 months
Mestais, ^127^	silicone	Pt	Subdural/ Epidural	+	-	-	Rhesus macaque	Nissl, Iba1, GFAP	26 weeks
Romanelli, ^125^	silicone	Pt	Subdural	+	-	+	Rhesus macaque	Nissl, Perls iron staining, Iba1, GFAP, Vimentin	30 weeks
Minev, ^128^	silicone with platinum-silicone composite coating	Au	Subdural	+	-	-	Lewis rats	Nissl, Iba1, GFAP	6 weeks
Ghanbari, ^139^	PMMA/PET	-	Epidural	-	+	-	GCaMP6f mouse	DAPI, GFAP	6,10,30 and 36 weeks

### Classical histological markers to show mechanical trauma

To understand the level of tissue reaction to mechanical trauma, the part of the extracted neuronal tissue that was in contact with the device earlier needs to be stained with histological markers. Both ipsilateral and contralateral sites (as controls) need to be investigated, both with low and higher magnification, for better comparison. Since biological tissues have little inherent contrast, histological stains are necessary to mark the region of interest in the cells to give further contrast to the tissue. Histological and histopathological staining are used to study both healthy and diseased tissue or to distinguish degenerating cells from healthy ones.

In this section, the most important methods that are widely applied in the literature as basic staining for labeling cell loss are listed. We discuss the availability of these methods used in recent literature to show mechanical trauma by the cortical depression of the ECoG device, cell loss, or neurodegeneration.

#### Nissl method

Nissl staining is one of the most well-known methods to quantify cell loss and neurodegeneration. The Nissl substance - rough endoplasmic reticulum - appears dark blue due to the staining of ribosomal RNA, giving the cytoplasm a mottled appearance. DNA present in the nucleus stains a similar color^126^.

Nissl staining is convenient for measuring the density of cells because stained cells are clearly defined and easily measured in all types of tissues. Nissl staining also shows cortical cytoarchitecture changes in cerebral cortical layers. Moreover, cellular loss caused by long-term mechanical trauma to the surrounding tissue, which can cause permanent damage in the superficial layers of the underlying cortical structures, can also be quantified. In Long Evans rats, a comparison of the cortical thickness of the underlying cortices at the ipsilateral vs. the contralateral site and the quantification of the level of cortical depression were examined 6 weeks following the implantation of an ECoG device. Histological results proved that brain depression greatly increased in the case of the implantation of epidural polyimide-based devices with nonuniform fenestration when compared to that of all other devices, conformable and nonconformable^123^. A wireless 64-channel WIMAGINE, ECoG recording implant was reported not to cause tissue reaction after 26 weeks of implantation in macaques and sheep (refs. ^122,125,127^). Newly developed organic devices have inherent elastic structures and, therefore, more flexible properties, causing less trauma to the surrounding structures. The cytoarchitectural analysis of the cortical tissue showed no or very small indentation of the tissue underneath subdurally implanted SWCNT-based ECoG arrays and electronic dura^124,128^.

#### Hematoxylin and eosin staining—H&E

Hematoxylin and eosin (H&E) staining is the gold standard of histopathological staining. It is the most widely used stain in medical diagnosis^129^. H&E is the combination of two histological stains: hematoxylin and eosin. Hematoxylin stains cell nuclei a purplish blue, and eosin stains the extracellular matrix and cytoplasm pink, with other structures taking on different shades, hues, and combinations of these colors^130^. H&E staining was used to establish whether collagen was present in the scar tissue to determine the tissue morphology^23^. An implantation area with normal leptomeningeal structures was also shown by H&E staining^121^. H&E staining showed minimal or no damage following the microsurgical protocol^131^. Following the 6-month implantation period, fibrosis, mild inflammatory cell infiltration, and mild angiogenesis were observed in the subcutaneous tissues surrounding the titanium case and silicone-covered coil unit, which are the normal foreign body responses to chronic implantation, and the subdural silicone-based Pt device was demonstrated to be biocompatible in the subcutaneous environment^132^.

#### Neuronal nuclei—NeuN

NeuN (neuronal nuclei) is a monoclonal antibody that recognizes a neuron-specific nuclear protein expressed by most neurons in the central and peripheral nervous system^133–135^. NeuN antibodies are the most important markers for detecting mature neurons. Unlike Nissl staining, NeuN does not stain glial cells, which offers a special approach for visualizing neurons separately from glia; therefore, NeuN staining can show neural density in neuronal tissues.

Neuronal density was found to be unchanged two months after the subdural implantation of an SWCNT/MDPE—Pt ECoG array; however, implantation might have caused mild neuronal damage because there was a trend in the percentage of NeuN+ cells harboring an abnormal phenotype^124^. GFAP (see 6.7) and NeuN staining was used to test for a potential increase in glial cell density and to compare viable neuron densities, respectively. After 3 weeks or 3 months, no significant damage was found based on GFAP and NeuN staining of the relevant brain areas after the epidural implantation of an extracellular matrix protein-coated (ECM-coated) polyimide substrate Pt device^136^. Neuronal density showed no significant difference between the implanted and control sites 6 months following implantation of the silicone/Pt ECoG electrode^132^.

#### Perls iron staining

Perls iron staining is a technique for the detection of the presence of iron in tissue that does not involve the application of a dye but causes pigment formation in the tissue. Perls iron staining reveals nonheme iron in tissues, such as ferritin and hemosiderin^137^. Perls iron staining revealed hemosiderin in the reactive tissue, suggesting an old hemorrhage in the areas of subdural implantation of a silicone-encapsulated Pt device^125^.

### Fluorescent histological markers for indicating mechanical trauma

#### DAPI - 4′,6-Diamidino-2-phenylindole and Hoechst dyes

DNA-specific fluorescent 4′,6-diamidino-2-phenylindole (DAPI) staining is an easy way to stain cell bodies^138^. The characteristic blue DAPI staining shows only nuclei of the labeled cells of the tissue.

Compared to those of the primary control, there was no significant difference in the intensity and number of cell nuclei stained with DAPI 80 days following epidural thio-lene/acrylate—Au device implantation^118^. No alterations in cortical layer structures were detected 3 days and 28 days following the epidural implantation of LCP-Au-Pt ECoG^114^. Cellular density changes were visualized and showed no changes using a transparent skull at every timepoint following surgery (6, 10, 30, and 36 weeks)^139^.

#### NeuroTrace Nissl stains

Dyes are proprietary; however, they are stains that label the Nissl substance, which is composed of ribosomal RNA associated with the rough endoplasmic reticulum and is present in high amounts in neuronal cells. The NeuroTrace Nissl dye exhibits fluorescence visible with fluorescein filters and is often more sensitive than cresyl violet dye (FluoroNissl Green dye^140^). Cellular density changes around the implanted ECoG in the cortical tissue can also be visualized by NeuroTrace dye^118^.

### Quantification of locally induced inflammation and immune response

**Fig. 4:**
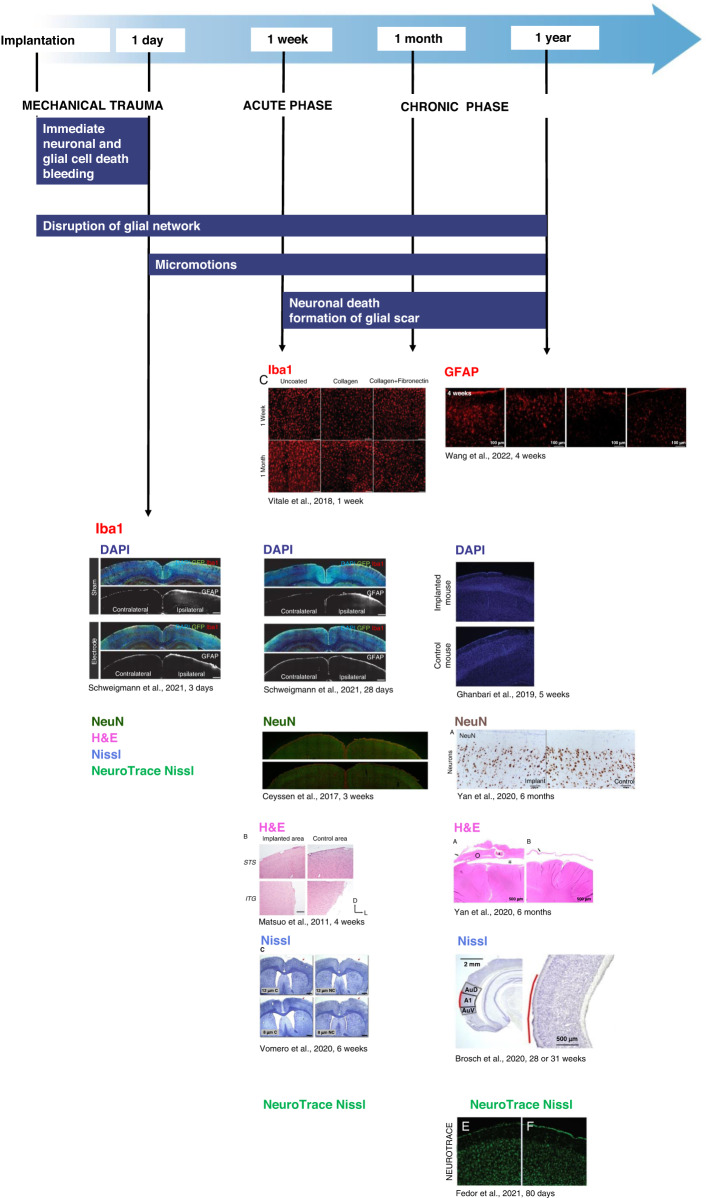
Evolution of glial scar formation. Examples of histological evidence for the biocompatibility of transparent neurointerfaces with the application of different staining methods. All possible applicable histological methods are listed; however, examples are shown only of those that were used in the literature. The images shown are from the cited articles with the specific survival times from each study. All figures are reproduced with permission

The damage caused by device implantation causes original trauma to the underlying tissues. Later, as the animal moves and functions, the permanent micromovement of the ECoG device, as a consequence of the relative stiffness of the ECoG device compared to that of the soft surrounding tissue, triggers chronic neuroinflammation. A few hours following implantation, activated (reactive) microglia migration to the injured environment is initiated, followed by reactive astrocyte activation a few days later. As time passes, the glial scar thickens, resulting in glial scar formation and encapsulation of the electrode (Fig. 4).

### Acute neuroinflammatory reaction—activated microglial response

The neuroinflammatory response is mediated by activated microglia. Activated microglia—the resident immune cells of the central nervous system—are quiescent microglia activated in cases of traumatic injury^141^. Early microglia activation following traumatic or focal brain injury can become chronic because of the micromovement of the implanted ECoG device. Such cells display a classically activated phenotype, releasing proinflammatory molecules, resulting in further tissue damage and potentially contributing to neurodegeneration.

#### Ionized calcium-binding adapter molecule 1—Iba1

Ionized calcium-binding adapter molecule 1 (Iba1), also known as allograft inflammatory factor 1 (AIF-1), is a protein encoded by the AIF1 gene. This gene has been shown to be highly expressed in microglia. The marker Iba1, which is upregulated in reactive microglia, is often used to visualize these cells in cases of brain injury.

Flexible ECoG devices usually do not facilitate the strong activation of microglia labeled by Iba1, even after a long survival period, either in the case of epidural (ref. ^23^ 1 year, ref. ^114^; 3 and 28 days, ref. ^107^; 28 or 31 weeks) or subdural (ref. ^124^ 2 months) implantation. The number of activated microglia beneath the hydrogel ECoG device was significantly less than that beneath the metal electrodes 2, 3, 4, and 14 weeks following subdural implantation^37^. ECM-coating was found to mitigate the activated microglial response, and the mean Iba1 pixel intensity at 1 week for the uncoated electrodes was significantly higher than that for the collagen-coated Parylene C/Au electrodes^142^. High-density subdural or epidural silicone/Pt ECoG device microglial responses were also evaluated by Iba1 staining, and no (ref. ^132^ 6 months, ref. ^127^ 26 weeks) or moderate (ref. ^125^; 30 weeks) neuroinflammation was found.

### Chronic neuroinflammatory reaction—reactive astrocytes

#### Glial fibrillary acidic protein—GFAP

Similar to the activated microglial reaction^141^, the characterization of reactive astrocyte activation and encapsulation is important in the long-term signal stability and impedance characteristics of ECoG devices. The resulting glial scar may have a toxic effect on local neurons and act as a physical barrier around the implant, thus insulating it from the remaining neurons and, in the case of an electrode, increasing its impedance^143,144^.

Glial cell activation is characterized by an increase in volume and processes (hypertrophy), an increase in cell number (proliferation), and inflammatory reactions, with increased production of intermediate filaments, particularly glial fibrillary acidic protein (GFAP)^145–147^. GFAP is a commonly used marker for evaluating reactive gliosis as an astrocytic reaction to injury. Implant-covered brain cortex areas are characterized by elevated GFAP intensity in glia limitans and in layer I of the brain cortex compared to normal cortical tissue far away from contact areas^122^.

In the case of epidurally implanted ECoG devices, the underlying neuronal tissue showed a significant increase in reactive astrocyte activation 1 year^23^, 3 days and 28 days^114^, 80 days^118^, 28 weeks, or 31 weeks^107^ following device implantation relative to the contralateral site. Coating extracellular matrix protein on polyimide-Pt devices improved biocompatibility; ECM-coated devices showed no significant GFAP reaction 3 weeks or 13 weeks following device implantation^136^. In the case of epidurally implanted high-density ECoG devices, the implant-covered brain cortex showed no loss in continuity for the glia limitans and reactive astrocytes. These findings revealed that the epidural implant did not damage the glia limitans nor induce astrogliosis in the cortex under the implant 26 weeks following the implantation of silicone-Pt^127^. Subdurally implanted ECoG arrays showed no differences in reactive astrocyte accumulation 2 months following device implantation; however, there was a strong trend toward an increase in GFAP staining on the ipsilateral side, suggesting the presence of mild reactive gliosis in the implanted cortex in the case of the SWCNT/MDPE/Pt ECoG array^124^. ECM-coating improved biocompatibility and protected neuronal tissue from scar formation 1 month after the subdural implantation of a Parylene C substrate Au device^142^. A high-density silicone/Pt-based ECoG implanted subdurally showed no GFAP accumulation after 6 months^132^. Implant-covered brain cortex areas showed a substantial increase in GFAP reactivity, extending from glia limitans to layer I of the cerebral cortex. Staining revealed that astrocytes became hypertrophic and elongated with thick processes compared to those in the intact brain region, which were more stellate in appearance. GFAP immunoreactivity was maximal at the periphery of the contact site of the subdural silicone-Pt/Ir-based ECoG array and declined progressively as a function of the distance from the periphery to the center of the contact area. Such elevation was observed up to 400 µm from the cortical surface^122^. Following device encapsulation, the reactive tissue surrounding the electrode array did not contain any GFAP-positive cells, indicating the absence of astrogliosis in the capsule and containing microglial and fibroblastic components only 30 weeks following subdural silicone/Pt ECoG implantation^125^. A hydrogel-elastomer—Ag/PVA neural interface resulted in much less glial reaction and cerebrovascular destruction compared to a metal electrode^37^.

### How to improve the histopathological characterization of transparent ECoG devices

After the euthanasia of animals, the explantation and removal of multiarray ECoG devices is one of the most critical steps and requires special attention (refs. ^122,127,132^). Skin and muscles can cover the implants, which need to be carefully removed. Device removal must be careful, and the tissue contact needs to be tested to determine whether any macroscopic signs of tissue defects, dural thickening, encapsulation, or changes in the vasculature below or around the device occurred.

It is necessary to check not only the electrochemical characteristics of ECoG devices but also the histopathological changes over time at the different timepoints of the experimental protocol. Simultaneous checking of impedance changes with snapshots of biocompatibility is necessary to better understand the mutual connections between recording or stimulating stability and biocompatibility. Encapsulation or glial scar formation is generally responsible for the increase in the impedance of the device in the long term^22,124^. Time presumably reduces the quality of the neural signal recorded by the ECoG array; however, stable recording can be achieved several weeks after implantation. The quality of the signals recorded on the first day of implantation up until four weeks after was further compared. Both recordings by the hydrogel and metal electrodes showed negligible changes over time^37^. The ECoG signals were reported as stable every day for 6 months in the case of wireless ECoG devices^125^.

Precisely following the electrochemical characteristics of devices with corresponding histopathological techniques can improve device construction and fabrication. Recently, surface modification techniques and coating materials have been proven to improve biocompatibility in the case of intracortical devices^148–150^ and polyimide- and Parylene C-coated ECoG devices^136,142^.

Nissl and H&E methods are the gold standards of histopathological staining. However, currently, fluorescent markers and conjugated primary antibodies are easily available and have fast staining protocols for fluorescence microscopy. Figure 4 shows classical and more recent histological techniques for indicating tissue damage and pathological neuroinflammatory responses at different timepoints following device implantation.

Straightforward visualization of the tissue environment of the device by brightfield or fluorescence microscopy is a very important issue. For the characterization of cellular loss, DAPI and NeuroTrace staining are easy fluorescent methods, whereas NeuN staining is the most feasible for visualizing selective neuronal loss. Iba1 is the gold standard for indicating acute neuroinflammation, whereas GFAP staining is the most commonly used staining method for indicating long-term glial scar formation.

To provide a general overview of the region of interest and the surrounding area, it is advisable to obtain a low-magnification image in which the site of device implantation (ipsilateral side) and the nonimplanted site (contralateral side) are both clearly visible for comparison. Additionally, obtaining a higher-magnification image of both the device implantation site and the control site is beneficial for comparing and estimating potential tissue damage, neurodegeneration, and the neuroinflammatory response of the implanted region relative to that of the intact region. The use of a sufficiently large magnification for visualization of the cell bodies of neurons, microglia, or astrocytes is important for better understanding and characterizing histopathological properties (ref. ^106^; Fig. 4).

## Application perspectives of multimodal, transparent neurointerfaces

The complementary nature of electrophysiology and fluorescence imaging in terms of spatial and temporal resolution is well recognized. Many studies utilize both methods simultaneously but not examining the same spatial location. The appeal of multimodal measurements has potently fueled the development of many different transparent devices with diverse material and technological approaches. Transparent ECoGs can record from large areas of the brain and thus extend the reach of high temporal sampling while remaining in the immediacy of imaging. Although the capabilities and unique benefits of novel devices are demonstrated upon their introduction, the possibilities of the combined methods are often left unexplored, and few are employed as a tool in experiments, despite thorough bench-top, in vitro, and in vivo testing. Here, we emphasize the advantages and potential uses of transparent ECoGs to promote their application in future multimodal experiments.

Although electrophysiology allows the recording of neuronal activity on a submillisecond scale^4^, it has a major limitation in its low spatial resolution due to the size of electrodes and the finite number of recording sites, especially with regard to its lack of visualization of specific neurons^18^. Imaging techniques allow these disadvantages to be overcome and provide a method for recording large populations of neurons with high spatial resolution using visual guidance. Despite the fact that the most recent imaging methods claim kilohertz functional imaging of single cells using voltage-sensitive dyes^151^, electrophysiology is still the gold standard for observing fast, functional responses of the nervous system. Therefore, the combination of electrophysiology recording and optical imaging could allow the temporal and spatial resolutions of these techniques to be taken advantage of, becoming a tool for mapping brain activity^9,3^.

Newly developed transparent devices^33,40,128,139^ and ECoG devices (refs. ^24,31,38,118,152^) are state-of-the-art technologies that combine these approaches.

The multimodal approach facilitated by the combination of imaging and electrophysiology yielded previously unattainable results in several areas. For example, it elucidated the correlations between subcellular function and cortex-wide activity. With the help of visual guidance, subcellular function was correlated with cortical surface activity. Two-photon imaging showed that dendritic calcium spikes appear at the cortical surface, proving their significance in the impact on surface potentials^153^, and helped to define the correlation between cortical dendritic activity and spindle-rich oscillations in sleeping rodents^154^.

In another case, it enabled the correlation of cell type-specific activity with areawise functional mapping. By the combination of electrophysiology and optical imaging, cortical barrel columns were mapped^155^, cortical glutamatergic neural activity during burst suppression was revealed^156^, and the interaction between stimuli-evoked cortical activity and low-frequency oscillation was examined^157^.

Last, the multimodal approach was beneficial for determining the role of nonneuronal cell types in health and disease. The importance of nonneuronal cell types, such as glial cells or immune cells, in the brain is well-known^158^. Electrophysiology has been fine-tuned for recording neuronal activity, and its technical arsenal is ill-equipped to monitor nonneuronal cell types. Moreover, imaging has long been used to monitor those cells along with neurons, which is particularly important in diseased states. For example, in an epilepsy model, epilepsy markers were identified by an ECoG, while two-photon imaging showed the role of glia–neuron interactions in responsiveness to sensory stimulation^159^. Another study showed the alteration of glia–neuron interactions during the transition from a preictal state to a generalized seizure, emphasizing a potential role for glia–glia and glia–neuron connections in the generation of epileptic seizures^160^. Furthermore, multimodal recordings were beneficial for determining the spatiotemporal evolution of ictal bursts in multiple epileptic models, demonstrating that the cerebellum is their main source^161^. Enlarging the scope of neurological disorders that can be readily studied by multimodal approaches, in a mouse model of Alzheimer’s disease, the simultaneous monitoring of astrocyte calcium and electric neuronal activities showed the impact of Aβ on sensory-evoked cortical activity^162^.

Concerning the improved spatial access facilitated by imaging, mesoscale or whole-brain imaging is becoming an increasingly popular method for investigating neuronal networks (refs. ^163–165^). Using either camera sensors or scanning solutions, fluorescent activity is gathered from large areas or the entirety of the rodent cortex, with state-of-the-art solutions capable of cellular resolution^166^. Similar to conventional implanted windows, bone over the field of view is replaced with glass or other transparent materials. As craniotomy covers a significantly larger area, this window is often custom-made and designed in consideration of the curvature of the brain^139,167^. This approach offers high imaging quality over a large field of view but with a great technical challenge. Thinning the skull to a thickness where transcranial fluorescent imaging is possible (10–50 μm) is a viable alternative to cranial windows^90,168,169^. The main advantages of this approach are avoiding the disturbance of the immune system and the possibility of imaging the same neuronal population over extended time periods at the cost of unhindered optical access and decreased proximity of ECoGs to the brain^170^. There are also several factors that hinder the widespread application of thinned skull preparations. First, the surgical expertise required for consistent, high-quality preparations is considerable. Second, the field of view is limited to ~0.1–0.3 mm^2^, a fraction of even conventional circular craniotomies, due to the risk of injury during surgery that could elicit an immune response and thus preclude one of the method’s main advantages. Finally, between imaging sessions, it is necessary to thin the skull again as the bone thickens and turns opaque over 1–2 days, which limits the number of imaging sessions to 3–5. Improvements to counteract these issues have been described: the size of transcranial windows and the consistency of thinning can be increased with automated surgical protocols, such as laser ablation^171^ or computer numerical control (CNC) milling^172^, and the transcranial window can be strengthened with glue or glass to prevent regrowth and preclude the need to rethin the bone^169^.

Transparent ECoGs may greatly improve mesoscale imaging experiments. Incorporating such devices into experimental design could facilitate the cortex-wide recording of local field potentials and oscillations in addition to imaging. There are two possible ways to achieve this with the cranial window technique. The first is to use transparent ECoG devices that cover the entire cortex together with cranial windows for mesoscale imaging. Here, tried-and-tested ECoG devices with established material combinations and fabrication processes could be readily used if the problems posed by implantation could be overcome. Devices covering the whole mouse cortex have already been demonstrated^173^, although with a suboptimal material composition for multimodality. Alternatively, recording sites and connection paths can be incorporated into the structural composition of the cranial windows. This approach could potentially provide electrophysiological information from the whole field of view, in addition to unrestricted optical access for imaging or neuromodulation. Such a device was demonstrated recently by Donaldson and colleagues^40^ by integrating ten PEDOT:PSS recording sites into inkjet-printed PET implants. This allowed the recording and correlation of calcium activity sampled from the entire cortex to brain-wide low-frequency oscillations and responses to whisker stimulation. It is possible to combine transparent ECoGs with thinned skull preparations, as demonstrated by Brodnick and colleagues^170^, who chronically recorded somatosensory evoked potentials (SSEP) through the thinned bone over 30 days with similar latency but smaller amplitude compared to epidural recordings. Similarly, through-skull optogenetic stimulations evoked smaller amplitudes but spatially distinct responses in acute experiments, which was attributed to scattering and difficult to compensate for or model due to the nonuniformity of the thinned surface.

Clinical diagnosis and therapy of epileptic human patients depend on recordings of seizures, interictal events, and pathological high-frequency oscillations^174^. Grid electrodes implanted sub- or epidurally are indispensable in determining the origin of seizures for resection surgery^175^. Scaled-down versions of ECoG grids are widely used in small animal experiments, which creates a common platform for interpreting the results of human measurements and animal experiments. Transparent ECoGs have the potential to extend the application range of this platform. Simultaneous measurements of fluorescence imaging, optogenetic modulation, and electrocorticography may allow the calcium activity of neuronal populations to be correlated with local field potentials and oscillations^42^. Correlating conclusions acquired from fluorescence imaging and optogenetic modulation with human measurements could thus extend our understanding in lieu of the fluorescent labeling of live human tissue.

Sharp waves are at the center of research interest for their principal role in memory. However, their study is hindered by the difficulties of accessing the hippocampus with optical methods. The removal of the overlaying cortex and implantation of an access window is now a widely used approach, but it makes electrophysiological access to the imaging area difficult. As a compromise, recordings are made distantly from the imaging site or contralaterally. There is currently a great effort to utilize transparent ECoGs in the study of sharp waves^39,176,43^. Liu and colleagues described transparent ECoGs implanted over the hippocampus for simultaneous electrophysiological and two-photon calcium imaging measurement of CA1 neurons. The ECoG grids allowed them to record sharp-wave ripples and characterize them into distinct categories as local—global and stationary—traveling^177,178^, while two-photon calcium imaging revealed several neuronal ensembles^179,180^. The transparency of the ECoG device allowed the identification of both findings in the same neuronal population simultaneously, revealing the connection that distinct ensembles are associated with different patterns of sharp-wave ripple events. Their work highlights the possibilities of transparent ECoGs and how simultaneous multimodal measurements of described phenomena can yield novel insights.

Liu and colleagues^43^ presented combined measurements of widefield calcium imaging and hippocampal electrophysiological recordings with a transparent electrode array. The devices used in the study were similar to conventional penetrating arrays, implanted in the hippocampus with recording sites arranged to measure from different layers. They exploited the transparency of the electrode arrays to perform optically unobstructed calcium imaging of the whole cortex, providing an advantage over existing flexible but nontransparent penetrating arrays. This approach allowed them to conclude that cortical activity, which showed distinct patterns, generally precedes hippocampal activity and that distinct cortical activity patterns are associated with different hippocampal population activities.

Interictal spikes are elusive events whose study could benefit greatly from the use of transparent ECoGs, as their mechanics and role in epilepsy are not fully understood^181,182^. The use of ECoGs in epileptic patients and animal models of epilepsy has increased the understanding of how seizures spread and which biomarkers offer accurate localization of seizure cores^6,183^. Transparent ECoGs could provide a method for adding to these findings in a similar way to that proposed for sharp waves. Interictal spikes are exclusively identified by their electrophysiological imprint. ECoG devices provide more information by spatially localizing events to specific cortical areas along with event time designation. Imaging of population or dendritic activity at that location is consequently correlated to a transient event with greater reliability than based on temporal co-occurrence with events recorded by distant electrodes.

## Future work

Simultaneous examination of neural networks through transparent ECoG devices provides an opportunity to better understand the physiological and pathological states of experimental animals and guide the application of these devices toward future human disease diagnostics and medication.

Despite great efforts and several successful proof-of-concept studies on the application of transparent neurointerfaces, there is still room for the investigation of these devices. The competing nature of this field produced novel technologies and materials that were not considered suitable for creating such microsystems before. These technologies and materials all relied on new concepts of device structures; however, there is a lack of extensive characterization, which is necessary before long-term in vivo use. Limitations of electrical, optical, and mechanical performance are seldom presented sufficiently. Only a few studies have monitored changes in the electrochemical properties of integrated features and the signal-to-noise ratio after implantation for a longer period of time^38,118^. Since these devices are placed directly in the beam path of complementary optical imaging techniques, the effect of the device on the optical resolution should be investigated. To date, a limited number of papers have addressed this issue by conducting experiments with fluorescent beads or labeled neuronal structures with and without the device to investigate the distortion of the optical image^9^. In addition to optical distortion, sensitivity to photoelectric artifacts is also rarely addressed. Defining the maximum laser intensity at various depths of the focal plane with respect to the device can be beneficial for determining how the contribution of electrical artifacts to the neuronal signals can be minimized^100^.

Proof of biocompatibility is a clear requirement when introducing novel devices for multimodal neuroimaging studies. On the other hand, not only could the device be harmful, but the conditions of optical imaging could also lead to the failure of these devices^9^. For example, high laser intensity used during deep-tissue scanning can damage the thin film layers of the device when used in the vicinity of the structural layer. To avoid this potentially harmful situation, device tolerance to excitation parameters should be determined. During device implantation, mechanical deformations (such as bending) can lead to device failure. Systematic mechanical tests should also be performed before moving on to testing surgical protocols^5,9,184^. The longevity and quality of optical signals detected through the transparent substrate and/or conductive layers should also be characterized in chronic in vivo use. Although these tests may seem too technical at first, they provide essential information to the final users, neuroscientists, to safely and fully exploit the advantages of multimodal investigations using transparent neurointerfaces.
